# The C2H2 zinc‐finger protein SlZF3 regulates AsA synthesis and salt tolerance by interacting with CSN5B

**DOI:** 10.1111/pbi.12863

**Published:** 2017-12-28

**Authors:** Ying Li, Zhuannan Chu, Jinying Luo, Yuhong Zhou, Yujing Cai, Yongen Lu, Junhui Xia, Hanhui Kuang, Zhibiao Ye, Bo Ouyang

**Affiliations:** ^1^ Key Laboratory of Horticultural Plant Biology, MOE, and Key Laboratory of Horticultural Crop Biology and Genetic improvement (Central Region), MOA Huazhong Agricultural University Wuhan Hubei China

**Keywords:** zinc finger, SlZF3, ascorbic acid, salt tolerance, ROS, CSN5B

## Abstract

Abiotic stresses are a major cause of crop loss. Ascorbic acid (AsA) promotes stress tolerance by scavenging reactive oxygen species (ROS), which accumulate when plants experience abiotic stress. Although the biosynthesis and metabolism of AsA are well established, the genes that regulate these pathways remain largely unexplored. Here, we report on a novel regulatory gene from tomato (*Solanum lycopersicum*) named *SlZF3* that encodes a Cys2/His2‐type zinc‐finger protein with an EAR repression domain. The expression of *SlZF3* was rapidly induced by NaCl treatments. The overexpression of *SlZF3* significantly increased the levels of AsA in tomato and *Arabidopsis*. Consequently, the AsA‐mediated ROS‐scavenging capacity of the *SlZF3*‐overexpressing plants was increased, which enhanced the salt tolerance of these plants. Protein–protein interaction assays demonstrated that SlZF3 directly binds CSN5B, a key component of the COP9 signalosome. This interaction inhibited the binding of CSN5B to VTC1, a GDP‐mannose pyrophosphorylase that contributes to AsA biosynthesis. We found that the EAR domain promoted the stability of SlZF3 but was not required for the interaction between SlZF3 and CSN5B. Our findings indicate that SlZF3 simultaneously promotes the accumulation of AsA and enhances plant salt‐stress tolerance.

## Introduction

Abiotic stresses, such as salt and drought stress, are a major cause of crop loss. Identification and functional characterization of the genes involved in stress tolerance is an important prerequisite for increasing the stress tolerance of crops. Previously, we performed a microarray analysis of salt‐stress responsive transcriptomes in tomato (Ouyang *et al*., 2007). We found that salt stress induces the expression of many genes that appear to encode transcription factors. One of these genes, *SlZF3* (GenBank Accession No. DY523809 and SGN Accession No. Solyc06g075780), encodes a putative ZAT12‐like Cys2/His2‐type (C2H2) zinc‐finger protein.

Zinc‐finger proteins are among the many transcription factors that promote stress tolerance. They are classified into several categories based on the number and the order of cysteine (C) and histidine (H) residues that contribute to the zinc‐binding activity of the zinc‐finger domains. C2H2 zinc‐finger proteins are one of the most prevalent types of zinc‐finger proteins in plants. Some zinc‐finger proteins carry an ethylene‐responsive element‐binding factor‐associated amphiphilic repression (EAR) motif, which can serve as an active repressor domain (Ciftci‐Yilmaz *et al*., 2007; Davletova *et al*., 2005; Hichri *et al*., 2014; Meissner and Michael, 1997). Many zinc‐finger proteins are important regulators of stress tolerance, such as ZAT12 from *Arabidopsis* (Davletova *et al*., 2005) and SlZF2 from tomato (Hichri *et al*., 2014).

Various stress treatments, including NaCl treatments, induce the expression of *ZAT12*. The overexpression of *ZAT12* improves osmotic stress tolerance in *Arabidopsis*. Null alleles of *ZAT12* reduce salinity and osmotic stress tolerance (Davletova *et al*., 2005). Similarly, *BcZAT12*, a ZAT12 homologue from *Brassica carinata*, enhances the drought tolerance of transgenic tomato plants (Chandra *et al*., 2012). ZAT12 and ascorbate peroxidase 1 (APX1) are key components of the network that scavenges reactive oxygen species (ROS) (Davletova *et al*., 2005; Rizhsky *et al*., 2004). Although ZAT12 appears to regulate APX1, this regulatory mechanism is not completely understood. In response to drought treatments, the activities of various antioxidant enzymes including APX are enhanced, which attenuates the accumulation of ROS. APX1 is a hydrogen peroxide‐scavenging enzyme that uses ascorbic acid (AsA) as the electron donor. The coexpression of ZAT12 with APX1 during abiotic stress treatments implicates this zinc‐finger protein in the regulation of AsA metabolism (Rizhsky *et al*., 2004).

AsA, as an important antioxidant, contributes to plant growth, development and stress responses by scavenging ROS produced as a by‐product of photorespiration (Hu *et al*., 2016; Smirnoff, 2000). Multiple pathways for AsA biosynthesis have been identified, including the D‐mannose/L‐galactose (D‐Man/L‐Gal), D‐galacturonate, D‐glucosone and myo‐inositol pathways (Davey *et al*., 1999; Loewus, 1999; Lorence *et al*., 2004; Wheeler *et al*., 1998). The D‐Man/L‐Gal pathway is particularly important in green plants. GDP‐Man pyrophosphorylase—known as GMP in tomato and VTC1 in *Arabidopsis*—is a key enzyme in this pathway. Indeed, AsA synthesis is decreased in *vtc1‐1*, an *Arabidopsis* mutant that is deficient in VTC1 activity (Conklin *et al*., 1999; Wang *et al*., 2013). When scavenging ROS, AsA can be oxidized by AsA peroxidase (APX) to yield monodehydroascorbate (MDHA). MDHA is further hydrolysed to generate dehydroascorbate (DHA). MDHA and DHA can be reconverted to AsA in the AsA recycling pathway catalysed by MDHA reductase (MDHAR) and DHA reductase (DHAR), respectively (Smirnoff and Wheeler, 2000).

Although the AsA biosynthesis and catabolism pathways are well established, the regulators of these important pathways are poorly understood. Indeed, only eight proteins were previously demonstrated to regulate AsA biosynthesis and in some instances, the regulatory mechanisms remain to be elucidated (Bulley and Laing, 2016). The GMP node appears to be important for regulating the accumulation of AsA. AtERF98 and SlHZ24 can bind to the promoter of the gene that encodes GMP and positively regulate its transcription (Hu *et al*., 2016; Zhang *et al*., 2012). CSN5B, a component of the photomorphogenic COP9 signalosome (CSN), can affect AsA biosynthesis and subsequently modulate plant responses to abiotic stress by binding to VTC1 and promoting its degradation in the dark (Wang *et al*., 2013). GMP activity is stimulated by KONJAC proteins (Sawake *et al*., 2015). AMR1 negatively modulates the expression of a few genes in the AsA biosynthetic pathway, but the underlying regulatory mechanism remains unknown. A variant of calmodulin, CML10, was shown to promote the accumulation of AsA by directly interacting with phosphomannomutase (Cho *et al*., 2016). We have a critical need to learn more about the factors that regulate AsA biosynthesis and metabolism for the purpose of both enhancing human nutrition by bio‐fortifying crops with AsA and increasing the tolerance of plants to abiotic stress.

Although there are nearly one hundred genes encoding C2H2 zinc‐finger proteins in the tomato genome, very few of them have been functionally characterized (Jin *et al*., 2014). Furthermore, there are no reports of zinc‐finger proteins affecting the regulation of AsA. Here, we report the functional identification of a novel gene named *SlZF3*, which encodes a C2H2‐type zinc finger. The expression of *SlZF3* was induced by salt stress. The overexpression of *SlZF3* increased the AsA content and enhanced the salt‐stress tolerance of tomato and *Arabidopsis*. We found that SlZF3 interacts with CSN5B, which negatively regulates AsA synthesis. Additionally, we found that SlZF3 and VTC1 competitively bind to CSN5B and that this competitive binding promotes the accumulation of VTC1. Consequently, the overexpression of *SlZF3* increased the AsA content and enhanced the salt tolerance of both tomato and *Arabidopsis*.

## Results

### *SlZF3* encodes a nuclear zinc‐finger protein

**Figure 1:**
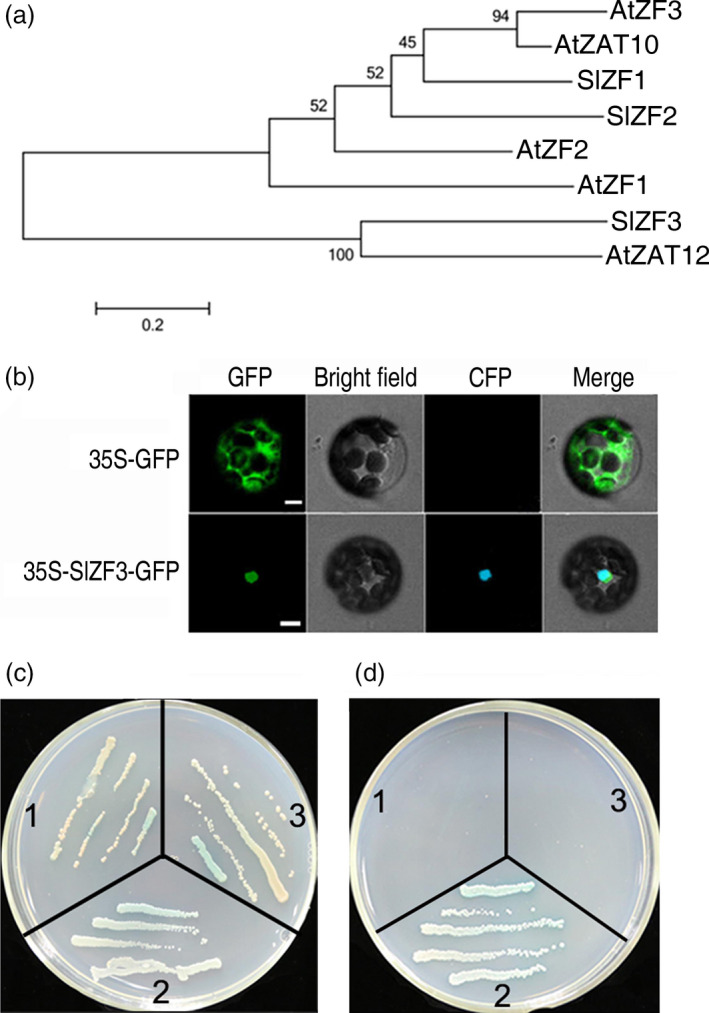
Characterization of tomato zinc‐finger protein SlZF3. (a) Phylogenetic relationships between SlZF3 (Solyc06g075780) and homologous zinc‐finger proteins from *Arabidopsis* and tomato. Scale bar, 0.2 amino acid substitutions per site. The bootstrap values from 1000 trials are indicated at the branch points. The phylogenetic tree was constructed using MEGA 5.1. (b) Nuclear localization of SlZF3‐green fluorescent protein (GFP) in *Arabidopsis* protoplast. Bars = 5 μm. (c–d) Transactivation activity of SlZF3. Cells were grown on selective media: SD/‐Trp/X‐α‐Gal and SD/‐Ade/‐His/‐Trp/X‐α‐Gal. The yeast cells were grown for 3–4 days at 30°C. 1, pGBKT7‐SlZF3 +  pGADT7‐EV (Empty vector); 2, positive control (cotransformation of pGBKT7‐53 carrying a fusion of GAL4 DNA‐BD and murine p53, and pGADT7‐RecT carrying a fusion of GAL4 AD and SV40 large T‐antigen); 3, negative control (pGBKT7‐Lam carries a fusion of the GAL4 DNA‐BD with human lamin C).

In our previous study, *SlZF3* (GenBank Accession No. DY523809 and SGN Accession No. Solyc06g075780) was identified as a salt‐inducible gene encoding a putative ZAT12‐like Cys2/His2‐type zinc‐finger protein (Ouyang *et al*., 2007). We isolated a full‐length cDNA clone of *SlZF3* with a length of 718 nucleotides that encodes a polypeptide containing 154 amino acid residues. SlZF3 is homologous to a variety of zinc‐finger transcription factors (Figure 1a). The amino acid sequence of SlZF3 is most similar to *Arabidopsis* ZAT12 (amino acid sequence similarity: 47.6%). The expression of ZAT12 is induced by many stresses, including NaCl, water deficit and abscisic acid (ABA) (Davletova *et al*., 2005; Soderman *et al*., 1996). Based on predictions from WoLF PSORT (Horton *et al*., 2007), SlZF3 contains two nuclear localization signal (NLS) sequences—KKRK and RRHR at positions 72, 97—and therefore may reside in the nucleus (Figure S1). Indeed, we found that both SlZF3 fused to the green fluorescent protein (GFP) and the nuclear marker protein GHD7 fused to the cyan fluorescent protein (CFP) were exclusively localized to the nuclei of protoplasts (Figure 1b). These data provide strong evidence that SlZF3 is a nuclear‐localized protein. To test whether SlZF3 can serve as a transcription factor, we performed a transactivation activity assay with SlZF3 in yeast cells. Unexpectedly, SlZF3 failed to activate the transcription of a GAL4 reporter gene in yeast (Figure 1c, d). However, the EAR motif located in the carboxyl terminus of SlZF3 (Figure S1) provides evidence that SlZF3 may serve as a transcription repressor in a suitable environment.

### Spatial and salt‐inducible expression of *SlZF3*

**Figure 2:**
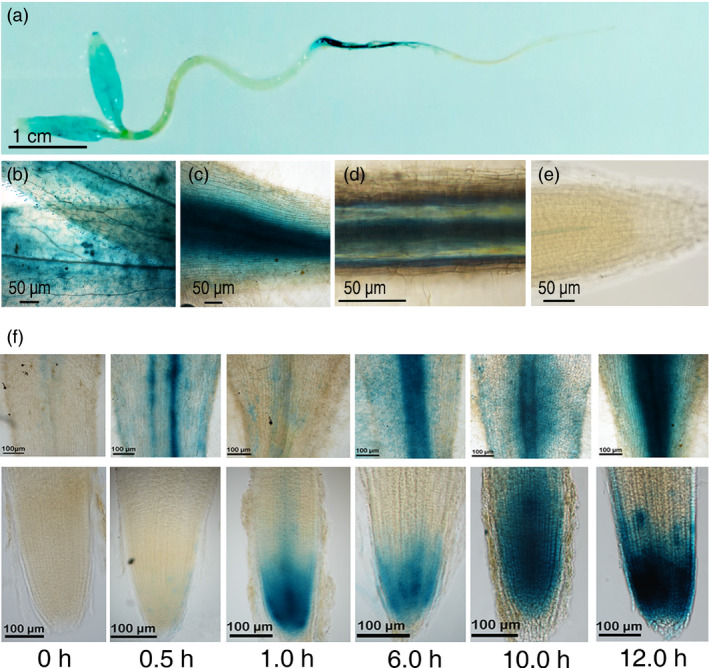
Spatial and salt‐inducible expression of *SlZF3*. (a) GUS staining of five‐day‐old transgenic seedlings expressing a *SlZF3* promoter‐driven GUS reporter gene. (b) cotyledon. (c) root/shoot junction. (d) root maturation zone. (e) root tip. (f) GUS staining following a 150 mM salt‐stress treatment in the root/shoot junction and root tip of two‐day‐old seedlings.

To study the expression of *SlZF3*, a 1.86‐kb promoter fragment from *SlZF3* was fused to the GUS reporter gene. Five‐day‐old transgenic seedlings were stained for GUS activity (Figure 2a–e). GUS expression was detected in cotyledons, stems, root‐shoot junctions and root tissue. These data indicate that this promoter is constitutively active in seedlings. Nevertheless, its expression in the root tip was much lower than in other tissues. Microarray analysis indicated that the expression of *SlZF3* is salt inducible (Ouyang *et al*., 2007). Therefore, we also analysed the expression pattern of *SlZF3* during a salt treatment that utilized 150 mM NaCl. Based on the intensity of the GUS staining, we observed that the expression of *SlZF3* was rapidly induced by this salt‐stress treatment (Figure 2f). Thirty min after the beginning of the salt treatment, we observed weak but visible GUS activity in the root tip. Thereafter, the GUS activity increased and reached a maximum after 12 h of salt treatment (Figure 2f).

### Constitutive expression of *SlZF3* improves salt tolerance in tomato

**Figure 3:**
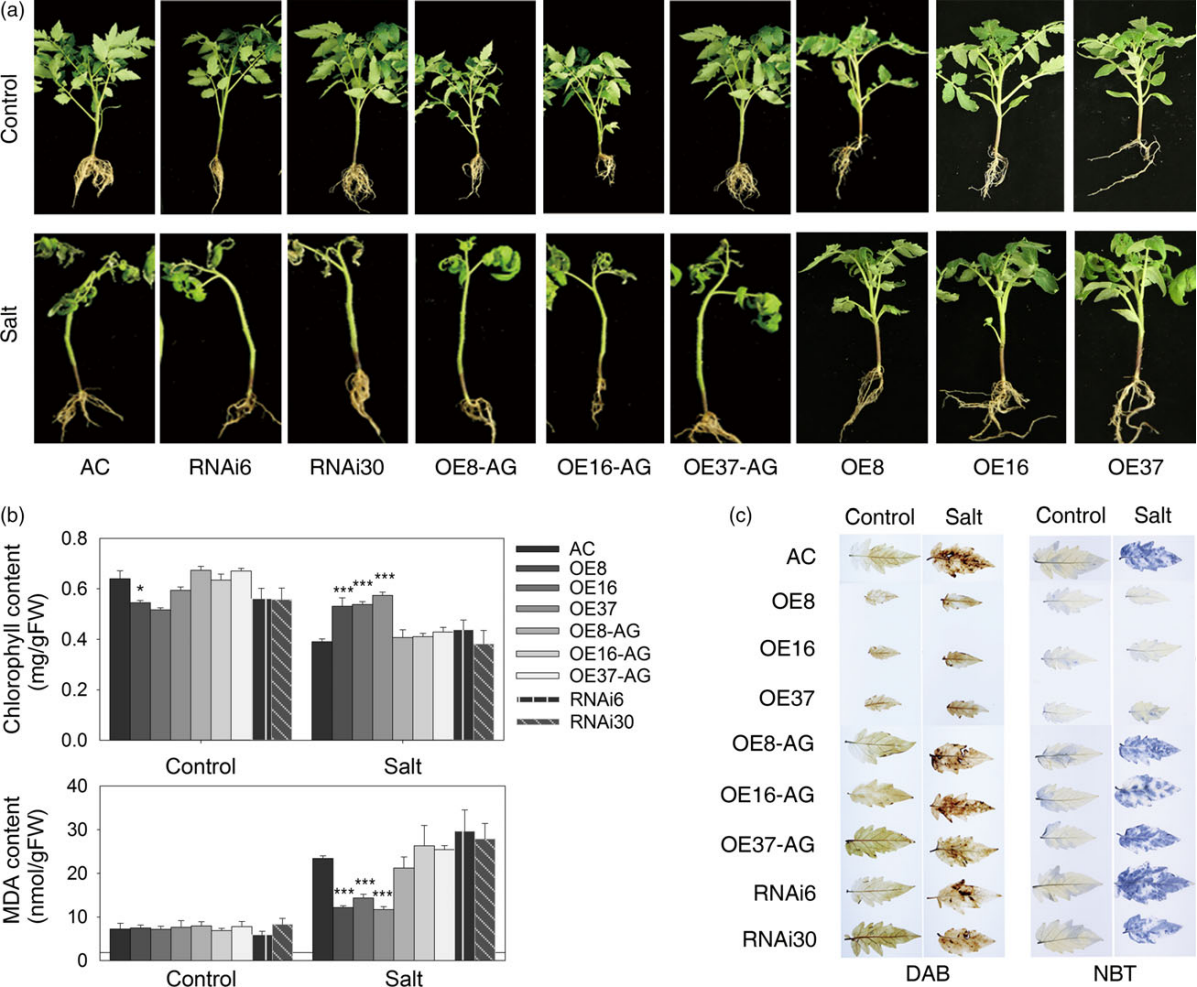
Overexpression of *SlZF3* enhances salt tolerance in tomato. (a) Phenotypes of wild‐type AC and transgenic lines grown under control conditions or 21 days of salt treatment. (b) The chlorophyll and MDA contents of tomato leaves. (c) The H_2_O_2_ and O_2_•^−^ contents determined by histological staining with DAB and NBT, respectively. AC, Alisa Craig; AG, azygous; OE, overexpression; RNAi, RNA interference. The asterisks indicate statistically significant differences in either chlorophyll or MDA contents between the transgenic plants and the wild‐type AC as determined by the two‐tailed Student's t‐test with equal variances (***P < 0.001; *P < 0.05). Data represent means and SD of three biological replicates.

After observing that the expression of *SlZF3* was induced by salt stress, we tested whether *SlZF3* affects salt tolerance. To test this idea, we generated three *SlZF3*‐overexpressing tomato lines (OE8, OE16 and OE37), two RNAi lines (RNAi6 and RNAi30) and three azygous lines (OE8‐AG, OE16‐AG and OE37‐AG) that segregated from their respective overexpression lines (Figure 3a). Under normal growth conditions in soil, the seedlings from the overexpression lines displayed a dwarf phenotype with limited root growth. In contrast, we observed no visible difference among wild type—Alisa Craig (AC)—and both the RNAi and azygous lines. Similar to AC and in contrast to the overexpression lines, the seedlings derived from the RNAi and azygous lines were sensitive to salt stress. Indeed, when the seedlings were irrigated with a salt solution for 3 weeks, the wild‐type seedlings were severely impaired. Specifically, they visibly wilted, developed chlorotic leaves and dropped leaves leaving only one to three leaves on the plant, and ultimately they died. These data demonstrate that the seedlings from the *SlZF3*‐overexpressing lines are significantly more salt tolerant than wild type (Figure 3a).

The effects of salt stress on tomato seedlings were also evaluated by quantifying their chlorophyll and malonyldialdehyde (MDA) content before and after the stress treatment (Figure 3b). Although under normal growth conditions, the chlorophyll levels in the overexpression lines were lower than the wild type, this relationship was reversed after 3 weeks of salt stress (Figure 3b). The chlorophyll levels in the stressed overexpression lines were significantly higher than those in the stressed wild type. The chlorophyll levels of the RNAi and azygous lines were similar to those of the wild type. The levels of MDA, an indicator of membrane damage, increased significantly in all of the materials tested. However, MDA levels were twofold to threefold higher in the wild type, RNAi and azygous lines relative to the overexpression lines (Figure 3b). We also examined the accumulation of hydrogen peroxide (H_2_O_2_) and superoxide (O_2_∙^‐^) radicals in leaves after salt stress by histochemical staining with 3, 3′‐diaminobenzidine (DAB) and photometric nitro blue tetrazolium (NBT). For DAB staining, although brown‐coloured polymeric oxidation products were visualized in all of the salt‐stressed tomato leaves, the intensity of the staining was markedly greater in the wild‐type AC, RNAi lines and azygous lines relative to the overexpression lines. Based on these data, we conclude that the overexpression of *SlZF3* reduces the levels of H_2_O_2_ that accumulate during salt stress (Figure 3c). The NBT staining experiment indicated that salt stress induced a significant increase in the accumulation of superoxide radicals in all of the tomato leaves that we tested with the exception of the overexpression lines, which exhibited no significant increase in NBT staining in response to salt stress (Figure 3c). Based on these data, we conclude that the overexpression of *SlZF3* reduces the levels of superoxide radicals that accumulate during salt stress. All of the above results demonstrate that overexpression of *SlZF3* promotes salt tolerance in tomato.

### SIZF3 interacts with CSN5B

**Figure 4:**
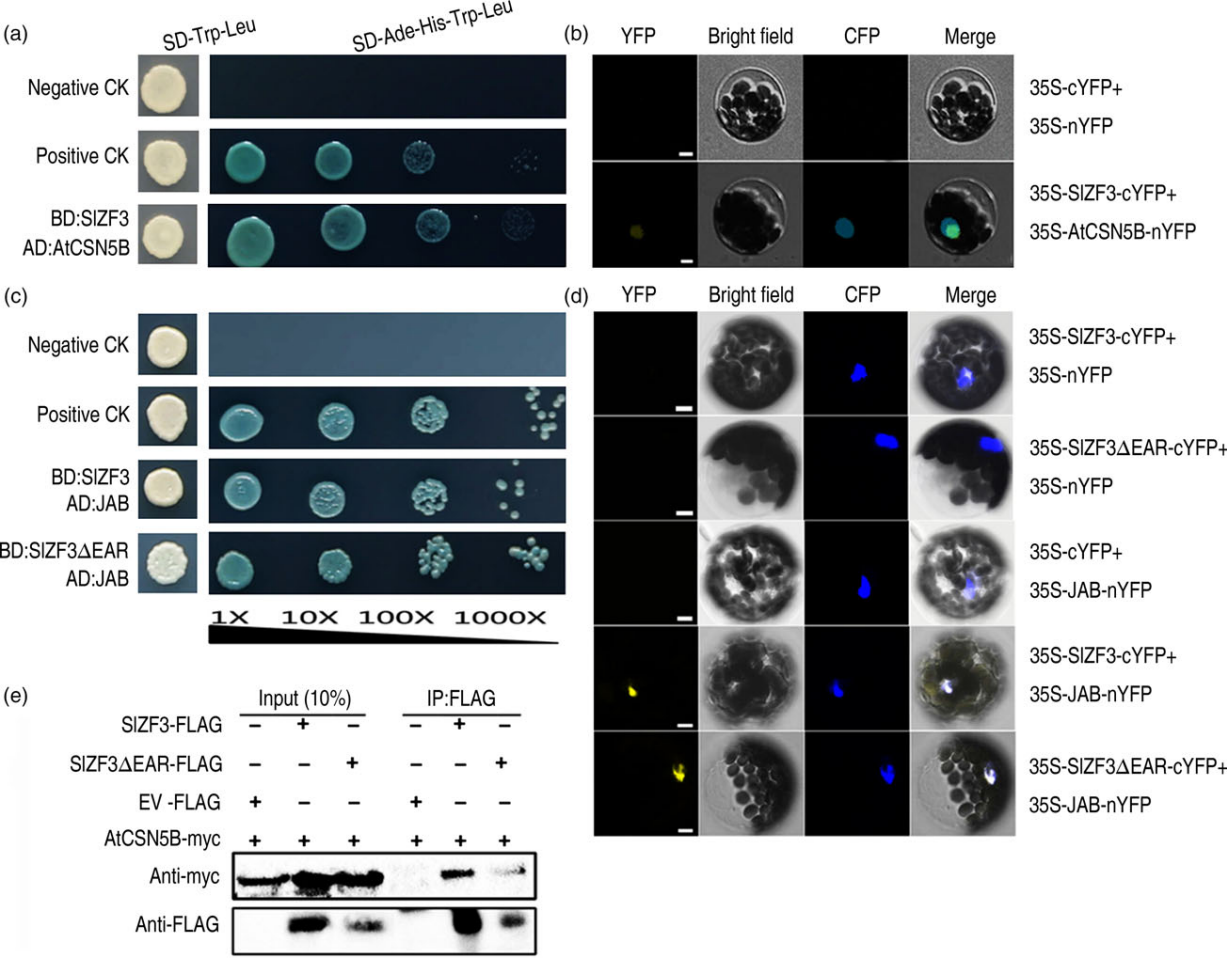
SlZF3 interacts with CSN5B *in vitro* and *in vivo*. (a, c) Yeast two‐hybrid assay demonstrating the interactions between SlZF3 and CSN5B or JAB. SlZF3 and SlZF3ΔEAR proteins fused to the GAL4 DNA‐binding domain (BD) were expressed in combination with CSN5B or JAB fused to the GAL4 activation domain (AD) in yeast strain AH109, respectively. Cells were grown on the following selective media: SD/‐Trp/‐Leu without X‐α‐Gal, and SD/‐Ade/‐His/‐Trp/‐Leu with X‐α‐Gal. pGBKT7‐53 + pGADT7‐RecT (positive control, pGBKT7‐53 carrying a fusion of GAL4 DNA‐BD and murine p53, and pGADT7‐RecT carrying a fusion of GAL4 AD and SV40 large T‐antigen); pGBKT7‐Lam+pGADT7‐RecT (negative control, pGBKT7‐Lam carries a fusion of the GAL4 DNA‐BD with human lamin C). Yeast strain AH109 cells were cotransformed with the above combinations of vectors and grown for 3–4 days at 30°C. (b, d) Bimolecular fluorescence complementation of YFP to test for interactions between SlZF3 with CSN5B/JAB in *Arabidopsis* mesophyll cell protoplasts. Bars = 5 μm. (e) SlZF3 interacts with CSN5B *in vivo*. SlZF3‐FLAG or SlZF3ΔEAR‐FLAG was transiently co‐expressed with CSN5B‐myc in tobacco (*N. benthamiana*) leaves using *Agrobacterium*‐mediated infiltration. Leaf tissue was harvested 2 days after infiltration. Total proteins were extracted from infiltrated leaves and used to perform immunoprecipitations with an anti‐FLAG affinity matrix. The resulting immunoprecipitates were assayed with anti‐myc and anti‐Flag antibodies to detect myc‐tagged CSN5B and Flag‐tagged ZF3/ZF3ΔEAR, respectively. EV (Empty Vector)‐FLAG was used as negative control.

To gain insight into the biochemical function of SlZF3, we performed a yeast two‐hybrid (Y2H) screen using SlZF3 as the bait and a prey cDNA library prepared from *Arabidopsis* plants. We sequenced a total of 96 positive clones. Ten of them encode CSN5B, a subunit of the COP9 signalosome (Table S1). Two experiments, including Y2H and bimolecular fluorescence complementation (BiFC), were performed to further test whether SlZF3 and CSN5B can interact. Our results demonstrated that SlZF3 interacts with CSN5B in both yeast and plant cells (Figure 4a, b).

We also tested whether SlZF3 interacts with JAB (the tomato homologue of CSN5B) using Y2H and BiFC. We found that SlZF3 directly interacts with JAB in yeast and plant cells (Figure 4c, d). We also tested whether the EAR motif in SlZF3 contributes to the interactions between SlZF3 and JAB by constructing a vector that expresses a deletion mutant of *SlZF3* in which the EAR motif is removed (SlZF3ΔEAR). We found that the EAR motif of SlZF3 was not necessary for the interaction between SlZF3 and JAB (Figure 4c, d).

Furthermore, to test whether the EAR motif contributes to the interaction between SlZF3 and CSN5B, we co‐expressed epitope‐tagged versions of either SlZF3 or SlZF3ΔEAR with an epitope‐tagged CSN5B in *Nicotiana benthamiana* leaves and then tested for protein–protein interactions by performing co‐immunoprecipitation (Co‐IP) assays with *N. benthamiana* leaf extracts. Specifically, either SlZF3‐FLAG or SlZF3ΔEAR‐FLAG was transiently co‐expressed with CSN5B‐myc in tobacco leaves. We found that CSN5B‐myc was captured in all of the samples and that CSN5B‐myc was co‐immunoprecipitated effectively with SlZF3‐FLAG. When the truncated SlZF3 lacking the EAR motif (SlZF3ΔEAR‐FLAG) was co‐expressed with CSN5B‐myc, we found that SlZF3ΔEAR‐FLAG was also co‐immunoprecipitated with CSN5B‐myc, although the Western blot signal was obviously weaker (Figure 4e). No signal was detected in the negative control (Figure 4e). Actually, the Western blot signal of SlZF3‐FLAG was markedly higher than that of SlZF3ΔEAR‐FLAG in both the input and Co‐IP samples (Figure 4e), which is consistent with the EAR motif promoting the stability of SlZF3.

### SlZF3 and VTC1/SlGMP competitively bind to CSN5B/JAB

**Figure 5:**
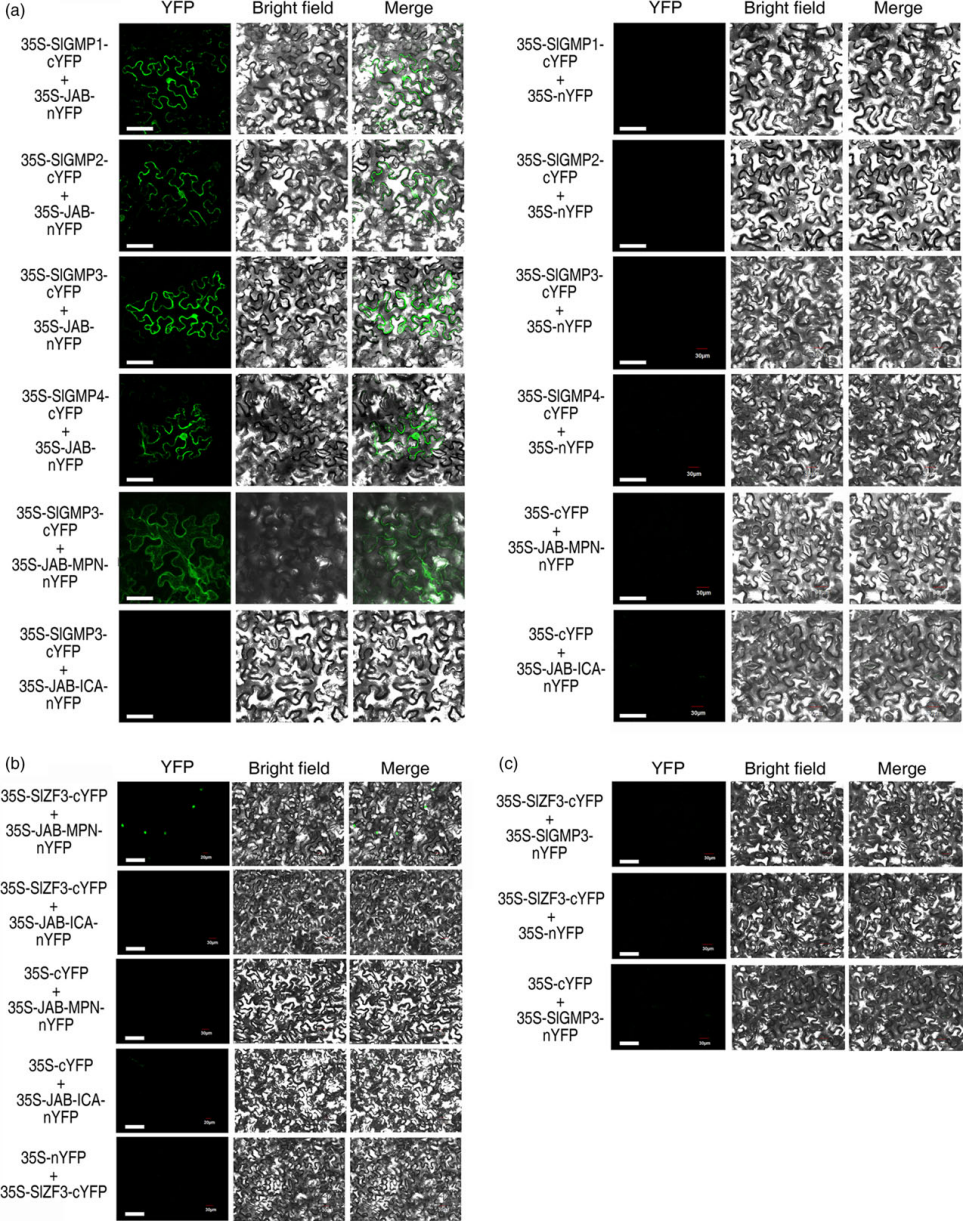
Interaction between SlGMPs and JAB, SlZF3 and JAB, and SlZF3 and SlGMP3. (a–c) The coding sequences of SlGMP1, SlGMP2, SlGMP3, SlGMP4 or SlZF3 without the stop codon were fused to the C terminus of yellow fluorescent protein (cYFP), and three different JAB fragments or SlGMP3 were fused to the N terminus of YFP (nYFP). Combinations of the above vectors and the empty‐vector control (35S‐cYFP, 35S‐nYFP) were transiently co‐expressed in tobacco leaves. YFP signals were detected by confocal laser scanning microscopy 48 h after infiltration. Bar = 50 μm.

We performed several experiments to assess the relationships among SlZF3, SlZF3ΔEAR, VTC1 (or SlGMPs) and CSN5B (or JAB). First, *Agrobacterium* infiltration‐based transient assays were employed to analyse the subcellular location of these proteins. We detected SlZF3 and SlZF3ΔEAR only in the nucleus. In contrast, VTC1, SlGMP1, SlGMP2, SlGMP3, SlGMP4, CSN5B and JAB were detected in both the nucleus and cytoplasm (Figure S2). Second, protein–protein interactions were analysed using BiFC. YFP signals were detected in both the nucleus and the cytoplasm when VTC1 and CSN5B were co‐infiltrated in tobacco leaves or either SlGMP1, SlGMP2, SlGMP3, or SlGMP4 and JAB were co‐infiltrated in tobacco leaves (Figures 5 and S3). For subsequent experiments, we used only SlGMP3 because SlGMP3 plays a major role in AsA biosynthesis relative to the other SlGMPs (Hu *et al*., 2016). We found that the VTC1 or SlGMP3 binding site on CSN5B or JAB is located in the MPN rather than the ICA domain (Figures 5a and S3). Third, we found that the SlZF3 binding site on JAB is also located in the MPN domain. These data are consistent with SlZF3 and SlGMP3 competing for the same binding site on JAB. In addition, we detected no direct interaction between SlZF3 and SlGMP3. These data provide evidence that SlZF3 does not directly regulate the SlGMP3 protein (Figures 5b, c and S3).

**Figure 6:**
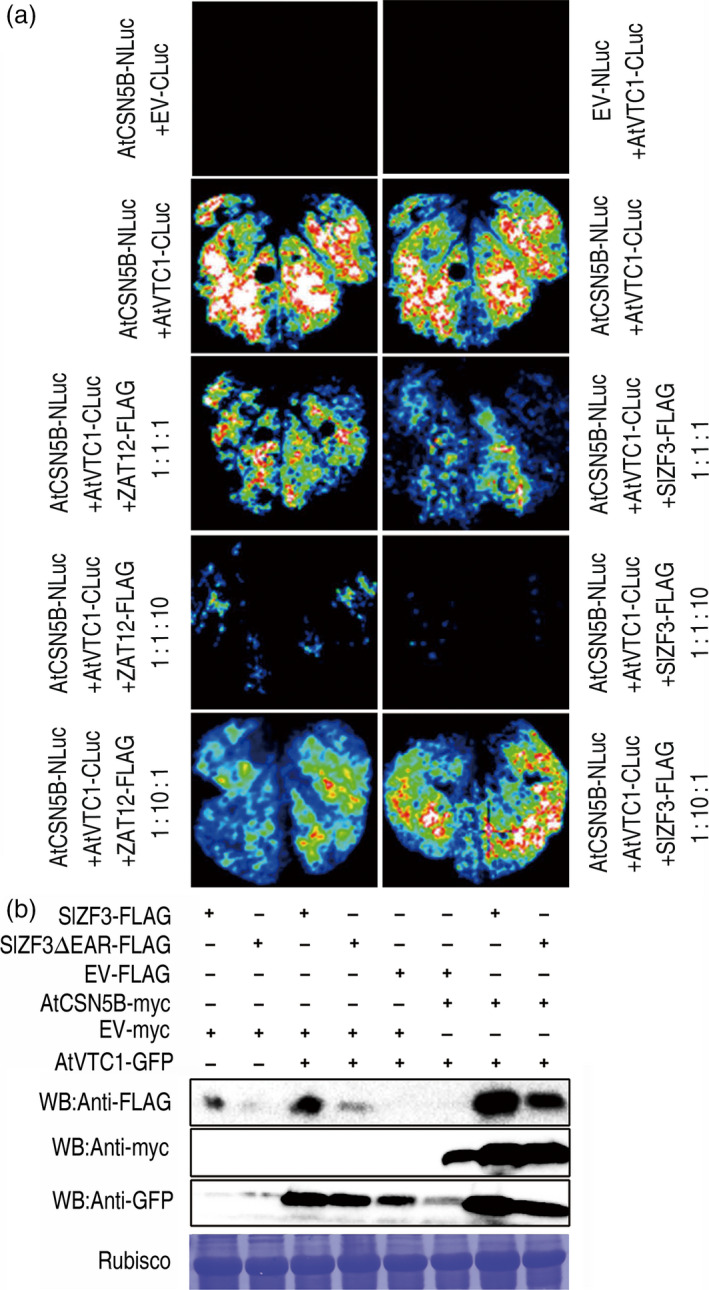
Competitive binding to CSN5B by VTC1 and SlZF3 in tobacco cells. (a) Images of transient expression assays in *N. benthamiana* leaves individually expressing either CSN5B fused to the N terminus of luciferase (CSN5B‐NLuc) or VTC1 fused to the C terminus of luciferase (VTC1‐CLuc) alone, or simultaneously expressing CSN5B‐NLuc and VTC1‐CLuc, or CSN5B‐NLuc and VTC1‐CLuc together with either 35S::ZAT12‐Flag (FLAG‐tagged ZAT12) or 35S::SlZF3‐Flag at ratios of 1:1:1, 1:1:10 and 1:10:1, respectively. (b) Influence of SlZF3, SlZF3ΔEAR, and CSN5B on the levels of VTC1. Combinations of SlZF3‐FLAG, SlZF3ΔEAR‐FLAG, CSN5B‐myc, VTC1‐GFP and the empty vector (EV‐FLAG, EV‐myc) were transiently co‐expressed in tobacco leaves. The tissue was harvested 2 days after infiltration, and total proteins of the infiltrated leaves were extracted and then analysed by immunoblotting with anti‐FLAG, anti‐myc and anti‐GFP antibodies.

Based on our data, we hypothesized that VTC1, a key enzyme in the production of the antioxidant ascorbate, and the zinc‐finger proteins SlZF3, SlZF3ΔEAR and ZAT12 (an *Arabidopsis* homologue of SlZF3) competitively bind to CSN5B. To test this idea, *Agrobacterium* infiltration‐based transient assays were performed with luciferase fragment‐tagged proteins (Figure 6a). When either CSN5B‐NLuc or VTC1‐CLuc was infiltrated into tobacco leaves, no bioluminescence was detected. However, we observed readily detectable bioluminescence when they were co‐infiltrated. These data indicate strong interactions between CSN5B and VTC1. Then, we performed a competition assay. CSN5B‐NLuc, VTC1‐CLuc, and either SlZF3‐Flag or ZAT12‐Flag were co‐infiltrated with a bacterial concentration ratio of 1:1:1 or 1:1:10 or 1:10:1. The luciferase signal was suppressed when CSN5B‐NLuc and VTC1‐CLuc were co‐infiltrated with either SlZF3‐Flag or ZAT12‐Flag. The signal was barely detectable when the bacterial solution of either SlZF3‐Flag or ZAT12‐Flag was infiltrated in a tenfold excess relative to CSN5B‐NLuc and VTC1‐CLuc. In addition, increasing the amount of VTC1 (1:10:1) induced an increase in the luciferase signal. These results indicate that both SlZF3 and ZAT12 compete with VTC1 for a binding site on CSN5B.

We performed immunoblotting to determine the levels of these proteins (Figure 6b). It is not surprising that VTC1 could be degraded by CSN5B (Wang *et al*., 2013). However, this degradation was inhibited when either SlZF3 or SlZF3ΔEAR was co‐expressed with VTC1 and CSN5B (Figure 6b). Therefore, both SlZF3 and SlZF3ΔEAR enhanced the accumulation of VTC1 when they were transiently co‐expressed with VTC1 in tobacco leaves (Figure 6b). Based on these data, we suggest that the competition between VTC1 and either SlZF3 or SlZF3ΔEAR for the same binding site on CSN5B inhibits the degradation of VTC1 by CSN5B.

### *SlZF3* enhances AsA biosynthesis and decreases H_2_O_2_ accumulation in tomato and *Arabidopsis*

**Figure 7:**
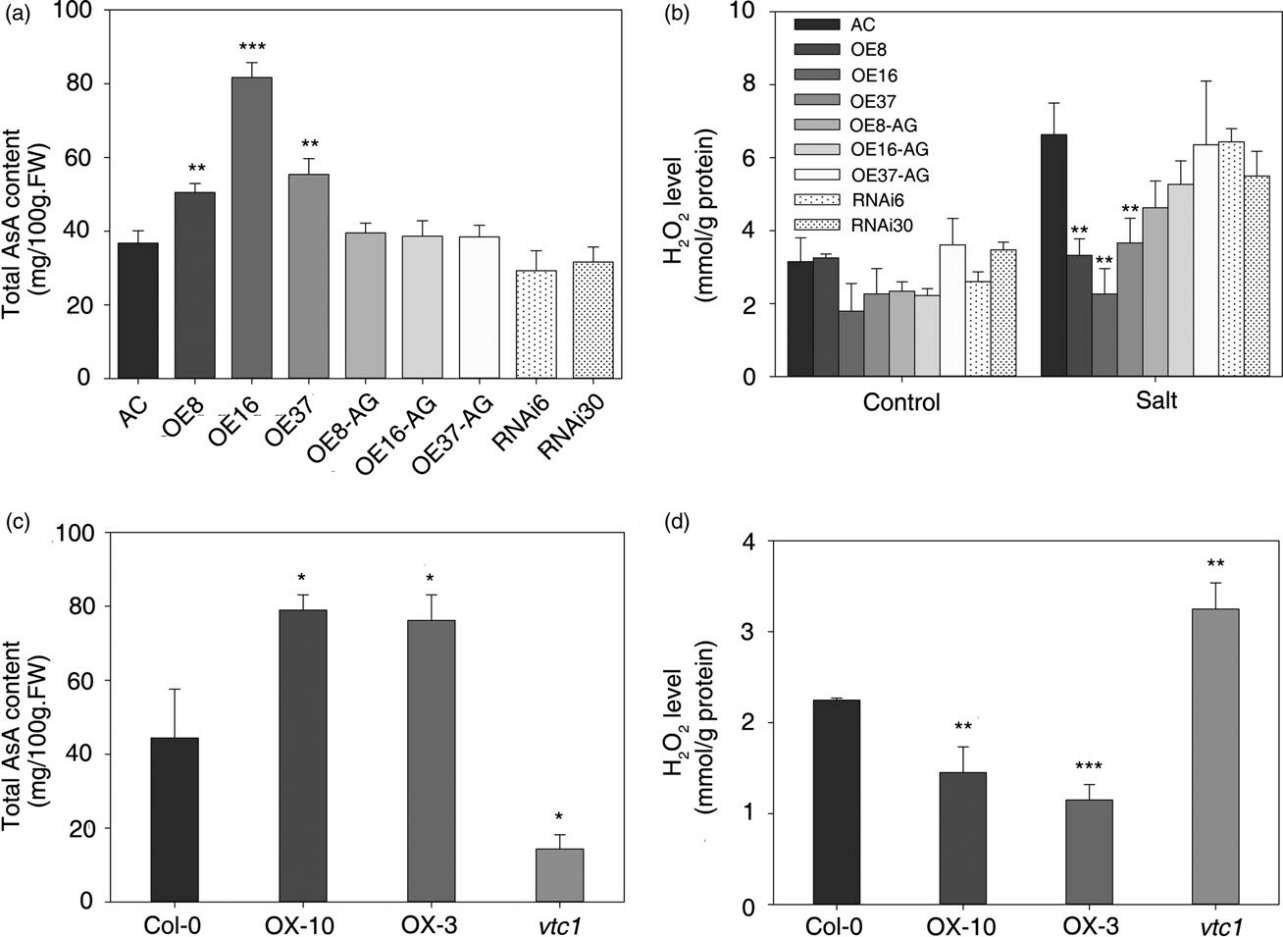
Total AsA content and H_2_O_2_ level in transgenic tomato and *Arabidopsis* leaves. (a, c) Total AsA content in tomato and *Arabidopsis*. (b, d) H_2_O_2_ content in transgenic tomato and *Arabidopsis* leaves. The asterisks indicate significant differences in the AsA and H_2_O_2_ content between the wild type (i.e. either AC or Col‐0) and either the transgenic lines or mutants. *P*‐values were calculated using the two‐tailed Student's t‐test with equal variances (***P < 0.001; **P < 0.01; *P < 0.05). Data represent means and SD of three biological replicates. AC, Alisa Craig; AG, azygous; OE, overexpression; RNAi, RNA interference.

CSN5B can affect AsA biosynthesis in *Arabidopsis* by binding to VTC1 and promoting its degradation (Wang *et al*., 2013). To test whether *SlZF3* is involved in the regulation of AsA biosynthesis, we analysed the AsA content in the transgenic lines of tomato. The AsA content increased significantly in the overexpression lines relative to the wild type, including a more than onefold change in OE16. The AsA content was decreased slightly in the RNAi lines but was not significantly different from the wild type and the azygous lines (Figures 7a and S4). Increased levels of AsA should affect the levels of H_2_O_2_, especially during salt stress. As expected, after 3 weeks of salt treatment, we observed significantly lower levels of H_2_O_2_ in the overexpression lines relative to the wild‐type plants (Figure 7b). Indeed, the salt treatment had no significant effect on the accumulation of H_2_O_2_ in the overexpression lines. In contrast, the salt treatment induced a more than onefold accumulation of H_2_O_2_ in the wild type, RNAi and azygous lines (Figure 7b).

To test whether *SlZF3* also affects AsA levels and salt tolerances in *Arabidopisis*, we created transgenic lines that overexpress *SlZF3* in *Arabidopisis*. The AsA content was analysed in wild‐type *Arabidopsis* (Columbia‐0), two overexpression lines (OX‐10 and OX‐3) and the *vtc1* mutant. We observed 78% and 72% increases in AsA levels in the two overexpression lines, respectively. In contrast, AsA levels were more than twofold lower in *vtc1* relative to the wild type (Figure 7c). As expected, we observed an inverse correlation between the levels of AsA and H_2_O_2_ (Figure 7c, d).

**Figure 8:**
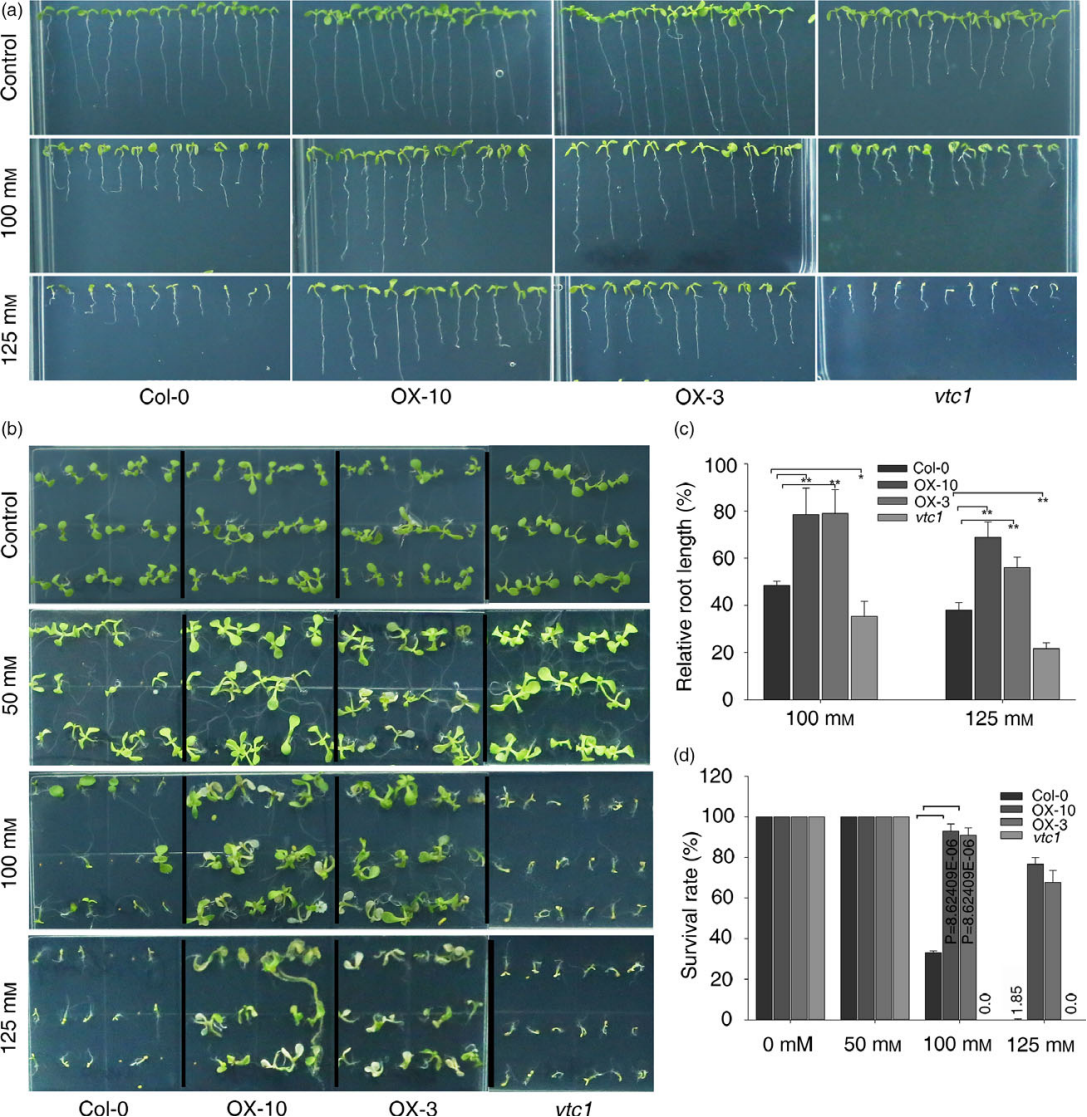
Overexpression of *SlZF3* enhances salt tolerance in *Arabidopsis*. (a, b) Phenotypes of wild type (Col‐0), transgenic lines and *vtc1* grown in 1% MS medium under normal conditions or with a salt (NaCl) treatment for 9 days. (c) Relative root length. The relative root length is the root length of the seedlings grown with salt relative to the root length of seedlings grown without salt. Each genotype was analysed in triplicate. Each replicate contained 12 plants. (d) Survival rate. Survival rates were determined in triplicate. Each replicate contained 18 plants. The asterisks indicate significant differences relative to the wild type (Col‐0). *P*‐values were calculated using the two‐tailed Student's t‐test with equal variances (**P < 0.01; *P < 0.05). Data represent means and SD of three biological replicates.

Our data indicate that the overexpression of *SlZF3* enhances AsA biosynthesis and reduces the accumulation of H_2_O_2_ in tomato and *Arabidopsis*. Similar to the tomato lines overexpressing *SlZF3*, we anticipated that the transgenic *Arabidopsis* lines that overexpressed *SlZF3* would exhibit enhanced salt‐stress tolerance. To test this idea, we challenged the transgenic *Arabidopsis* plants with NaCl both in MS agar medium (Figure 8) and compound soil (Figure S5). Root length was measured using vertically grown seedlings after 9 days of salt treatment. The relative root lengths of seedlings grown in MS media containing 100 and 125 mM NaCl were significantly longer for the two overexpression lines and significantly shorter for the *vtc1* mutant (Figure 8a, c). When horizontally grown on MS medium containing a low concentration of NaCl (i.e. 50 mM), the survival rates were not significantly different among the various genotypes (Figure 8b, d). However, under 100 mM NaCl, more than 90% of the overexpression lines survived, only 33% of the wild‐type seedlings survived and all of the *vtc1* seedlings died (Figure 8b, d). With 125 mM NaCl stress, the survival rates were reduced to 67%, 76% and 1.85% for OX‐3, OX‐10 and wild type, respectively (Figure 8b, d).

As expected, when grown in soil, tolerance to the salt treatment was significantly enhanced in the *SlZF3‐*overexpressing lines and significantly reduced in *vtc1* relative to the wild type (Figure S5a). After 2 weeks of salt treatment, the relative fresh weight and chlorophyll content were significantly higher in the two overexpression lines and significantly lower in the *vtc1* mutant relative to the wild type (Figure S5b, c).

## Discussion

Here, we report that *SlZF3*, a ZAT12‐like C2H2‐type zinc‐finger protein from tomato, and VTC1 competitively bind to CSN5B and that this competition inhibits the degradation of VTC1. Consequently, we observed that overexpressing *SlZF3* induced increases in the AsA content and enhanced the salt‐stress tolerance of both *Arabidopsis* and tomato. Additionally, although we found that this motif promotes the stability of SlZF3, this motif did not contribute to the CSN5B binding activity of SlZF3.

### Zinc‐finger protein functions as a new regulator for ascorbate biosynthesis

Ascorbic acid plays important roles in plant growth, development and stress responses (Hancock and Viola, 2005; Smirnoff and Wheeler, 2000). Although AsA biosynthetic pathways are well established, there are major gaps in our understanding of the mechanisms that regulate AsA biosynthesis. Previously, a total of eight proteins were reported to regulate the biosynthesis of AsA at the level of transcription or translation (Bulley and Laing, 2016). Four of these proteins can regulate GDP‐Man pyrophosphorylase (GMP). AtERF98 and SlHZ24 can bind to the promoter of the *GMP* gene and activate its transcription (Hu *et al*., 2016; Zhang *et al*., 2012). The COP9 signalosome subunit CSN5B promotes the degradation of VTC1 (the *Arabidopsis* ortholog of GMP) through the 26S proteasome in the dark (Wang *et al*., 2013). The KONJAC proteins promote the accumulation of GMP and act as positive regulators of AsA biosynthesis (Sawake *et al*., 2015). There are four additional proteins that contribute to the regulation of AsA. Although AMR1 is known to act as a negative regulator of AsA biosynthesis, the mechanistic details of AMR1‐mediated regulation of AsA biosynthesis are not known (Zhang *et al*., 2009). CML10 promotes the accumulation of AsA using a mechanism that involves interacting with phosphomannomutase (Cho *et al*., 2016). Although both VTC3 and CSN8 were reported to be involved in the regulation of AsA content, the underlying mechanisms remain unknown (Conklin *et al*., 2013; Wang *et al*., 2013).

**Figure 9:**
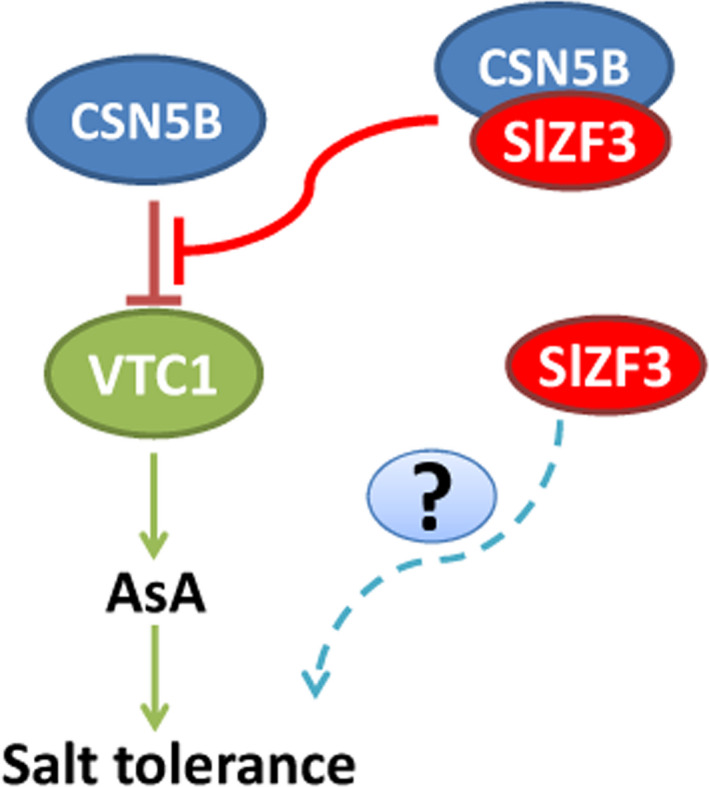
Model for the promotion of AsA accumulation and salt tolerance by SlZF3. CSN5B physically interacts with GDP‐Man pyrophosphorylase (VTC1) and promotes its degradation through the 26S proteasome pathway. SlZF3 and VTC1 competitively bind to CSN5B. This competitive binding inhibits the degradation of VTC1 by CSN5B. VTC1 promotes the biosynthesis of ascorbic acid (AsA). Consequently, SlZF3 promotes the accumulation of AsA and plant salt tolerance by enhancing the reactive oxygen species scavenging capacity of plants. Besides, SlZF3 may also regulate other factors to improve salt tolerance.

Our results from a variety of experiments indicate that SlZF3 is a novel regulator of the CSN‐VTC1 pathway. Specifically, we found that SlZF3 and VTC1 competitively bind to CSN5B and that this competition prevents CSN5B from targeting VTC1 for degradation through the 26S proteasome (Figure 9). Y2H screening indicated that CSN5B and SlZF3 can interact (Figure 4). CSN5B serves as an important regulator of AsA biosynthesis by degrading VTC1, the *Arabidopsis* GMP in the AsA pathway (Wang *et al*., 2013). To investigate the possibility that these proteins might interact *in vivo*, we determined the subcellular location of SlZF3 and SlZF3ΔEAR, CSN5B and JAB (a CSN5B homologue from tomato), and VTC1 and its tomato homologues (SlGMP1, SlGMP2, SlGMP3 and SlGMP4), and we found that all of these proteins accumulate in the nucleus (Figure S2). Further, we carried out BiFC experiments to test their interactions. We found that both SlZF3 and SlZF3ΔEAR interact with both CSN5B and JAB. We also found that CSN5B and JAB interact respectively with VTC1 and SlGMP3, which make the greatest contribution to AsA biosynthesis (Hu *et al*., 2016). However, we were not able to detect interactions between SlZF3 and SlGMP3 (Figure 5c). Further, both SlZF3 and VTC1/SlGMP3 bound to the MPN domains of CSN5B and JAB, which is consistent with SlZF3 and VTC1/SlGMP3 competitively binding to CSN5B and JAB (Figures 5, S3 and S4). Finally, using *Agrobacterium* infiltration‐based LUC bioluminescence transient assays and Western blotting analysis, we found that SlZF3 and VTC1 competitively bound to CSN5B (Figure 6a, b). It is highly probable that this mechanism is conserved in *Arabidopsis* and tomato. It is also possible that other zinc‐finger proteins use a similar mechanism to promote abiotic stress tolerance. For instance, recent research from our colleague revealed that an ATL78‐like RING‐H2‐finger protein (ShATL78L) can interact with CSN5B in tomato and that overexpressing this protein can enhance the tolerance of tomato to multiple abiotic stresses (Song *et al*., 2016).

As there is an EAR motif in the carboxyl terminus of SlZF3, it may act as a transcriptional repressor and directly regulate the transcription of genes that contribute to the accumulation of AsA. To test this, we quantified the expression of genes involved in the biosynthesis of AsA and the regulation of the biosynthesis of AsA (i.e. *JAB* and *CSN5B*) in the *SlZF3*‐overexpressing tomato and *Arabidopsis* lines (Figure S6). Our qRT‐PCR results showed inconsistent changes in expression of AsA‐related genes in the two *SlZF3*‐overexpressing tomato lines. The transcript of many genes declined in OE8, but they showed no significant change in OE16 (Figure S6a). In our transgenic *Arabidopsis* lines, the expression of most AsA‐related genes did not change significantly (Figure S6c). At the transcriptional level, no obvious change could be detected for *JAB* and *CSN5B* between the overexpression lines and the wild type (Figure S6b, d). Although the expression of some genes changed, these changes could not explain the up‐regulation of AsA in the overexpression lines.

To sum up, SlZF3 regulated AsA levels by regulating the stability of SlGMP/VTC1. We found that SlZF3 and SlGMP/VTC1 competitively bind to JAB/CSN5B and that this competition protects SlGMP/VTC1 from degradation by JAB/CSN5B, a key component of the CSN complex. Thus, SlZF3 promotes the accumulation of AsA by promoting the accumulation of SlGMP or VTC1 and therefore serves as a novel regulator of the CSN‐VTC1 pathway. A previous report showed that ZAT12 is indispensable for *Apx1* expression during oxidative stress (Rizhsky *et al*., 2004). However, in *ZAT12*‐overexpressing lines, *APX1* mRNAs do not accumulate relative to the wild type, which is consistent with an additional factor contributing to the regulation of *APX1* expression (Davletova *et al*., 2005). Our results indicated that CSN5B may serve as this factor and that CSN5B may fill the gap in the model proposed by Rizhsky *et al*. (2004) by affecting the stability of AsA biosynthetic enzymes, such as SlGMP/VTC1, and consequently affecting the expression of APX1.

### The EAR motif and CSN5B could affect the stability of SlZF3

Our results showed that the EAR motif was not necessary for the protein–protein interactions between SlZF3 and CSN5B and that the EAR motif is important for the stability of SlZF3 (Figures 4 and 6). In *Arabidopsis,* there are at least 20 zinc‐finger proteins that possess the EAR motif (Le *et al*., 2016). The consensus sequences L/FDLNL/F(x)P defines the EAR motif (Kagale and Rozwadowski, 2011). A previous study has shown that TOE1 (TARGET OF EAT1) is a member of the APETALA2 (AP2) family with an EAR‐like motif that does not appear to affect the protein–protein interactions between TOE1 and CO. However, transgenic plants overexpressing TOE1ΔEARL still flower earlier than wild type, which indicates that the EAR‐like motif is required for TOE1 to repress flowering (Zhang *et al*., 2015). Nevertheless, it is not clear whether the EAR‐like motif affects the stability of TOE1. It is also known that the EAR‐like motif makes important contributions to the activities of ZAT7 and ZAT12 and that it is required for protein–protein interactions (Ciftci‐Yilmaz *et al*., 2007; Le *et al*., 2016). Additionally, the EAR motif contributes to the activity of a C2H2‐type zinc‐finger protein named PtiZFP1 that interacts with MAPKs (Hamel *et al*., 2011). In our Co‐IP experiment, the input fraction for all of the samples was equal, but SlZF3 accumulated to significantly higher levels than SlZF3ΔEAR (Figure 4e). The enhanced accumulation of SlZF3 relative to SlZF3ΔEAR was independently verified by Western blotting (Figure 6b). Therefore, our results showed that although the EAR motif was not necessary for the association of SlZF3 and CSN5B, it was still important for the stability of SlZF3.

Additionally, CSN5B could also affect the stability of SlZF3. Our data from Western blotting indicated that when SlZF3 or SlZF3ΔEAR and CSN5B were transiently co‐expressed in tobacco leaves, the accumulation of SlZF3 and SlZF3ΔEAR was induced (Figure 6b).

### Overexpression of *SlZF3* enhances salt‐stress tolerance by increasing the levels of AsA

Accumulating data indicate that AsA can enhance the tolerance of plants to various abiotic stresses (Hemavathi *et al*., 2010; Le *et al*., 2016; Wang *et al*., 2013; Zhang *et al*., 2012). One important role of AsA is to scavenge ROS, which accumulate in response to various types of stress in plants (Hu *et al*., 2016; Li *et al*., 2010; Smirnoff and Wheeler, 2000). Our results showed that AsA accumulated to elevated levels in the *SlZF3* overexpression lines of tomato and *Arabidopsis* and that the levels of H_2_O_2_ were lower in these lines after a salt‐stress treatment (Figures 3c and 7). Based on these data, we conclude that the overexpression of *SlZF3* enhanced the salt tolerance of tomato and *Arabidopsis* (Figures 3 and 8). Interactions between various zinc‐finger proteins and CSN5B may universally improve stress tolerance by promoting the accumulation of AsA. To verify this, a role for AsA in the enhanced stress tolerance promoted by SlZF2 (Hichri *et al*., 2014) and ShATL78L (Song *et al*., 2016) should be tested. The *SlZF3*‐overexpressing plants displayed a dwarf phenotype that restricts its usefulness for the genetic improvement on abiotic stress tolerance. However, this problem might be resolved by addressing the mechanism of dwarfism or using suitable stress‐inducible promoters (Kasuga *et al*., 1999).

Although we provide compelling evidence that the enhanced accumulation of AsA and ROS‐scavenging capacity of the overexpression lines enhances their abiotic stress tolerance, we cannot exclude the possibility that the enhanced abiotic stress tolerance of these overexpression lines is a complex trait. For example, although we obtained 10 clones that encode CSN5B from our Y2H screen, we also obtained 13 clones that encode an ATPase and five clones that encode RD21a. Both ATPase and RD21a could potentially contribute the salt tolerance (Gévaudant *et al*., 2007; Guo *et al*., 2009; Vitart *et al*., 2001). More work is required to understand the mechanism of enhanced salt tolerance in *SlZF3*‐overexpressing plants.

## Conclusions

Our data demonstrated that SlZF3 and VTC1 competitively bind to CSN5B and that this competition attenuates the negative regulation of VTC1, a key AsA biosynthetic enzyme. Overexpressing *SlZF3* in tomato and *Arabidopsis* induced the accumulation of AsA, which relieved the oxidative damage caused by salt stress by enhancing the ROS‐scavenging capacity of the transgenic plants. The EAR motif appears to promote this mechanism by stabilizing SlZF3. We propose a working model for the promotion of AsA levels by SlZF3 (Figure 9). This work may lead to the biofortification of crops with AsA, especially with the optimization of spatiotemporal expression of zinc‐finger proteins using inducible or tissue‐specific promoters.

## Experimental procedures

### Gene isolation and the generation of transgenic lines

A salt‐inducible gene named *SlZF3* (GenBank Accession No. DY523809, SNG Accession No. Solyc06g075780) which encodes a zinc‐finger protein was identified in our previous study (Ouyang *et al*., 2007). The 513‐bp cDNA, a 216‐bp cDNA fragment and the 1.86‐kb promoter region of *SlZF3* were amplified from cDNA or genomic DNA, respectively, using specific primers (Table S2). The PCR products were cloned into pCR8/GW/topo (Invitrogen, Carlsbad, CA) and subsequently recombined into the plant expression vector pK2GW7 for overexpression of cDNA, pK7GWIWG2(II) for RNA interference using a cDNA fragment, or pKGWFS7 for promoter analysis with the GUS reporter gene (Karimi *et al*., 2002) using LR recombination reactions (Gateway™ technology, Invitrogen). The constructs were introduced into *Agrobacterium tumefaciens* strain GV3101 by electroporation and then into tomato (Alisa Craig) using the leaf disc method (Ouyang *et al*., 2005). The overexpression vector was also introduced into *Arabidopsis thaliana* ecotype Columbia (Col‐0) using the floral dip method (Clough and Bent, 1998). Homozygous transgenic *Arabidopsis* lines (OX3, OX10) with single T‐DNA insertion site were obtained by screening seedlings on MS medium with kanamycin for two successive generations. Homozygous transgenic tomato lines with single T‐DNA insertion sites were obtained as described in our previous paper (Ouyang *et al*., 2005).

### Plant materials, growth conditions and stress treatments

Wild‐type *Arabidopsis* (Col‐0), transgenic lines and *vtc1* (kindly provided by Dr. Rongfeng Huang) were grown under white light (3500 Lux) with a 16‐h light/8‐h dark cycle at 21 ± 2°C. The growth conditions for tomato were similar to that for *Arabidopsis*, except that tomato plants were grown at 25°C. For the salt treatments of tomato, plants from all lines were analysed with 12 replicates per line and eight individuals per replicate. The tomato plants were randomly grown in a tray (40 × 80 × 10 cm) filled with vermiculite and soil mixed in a 1:1 ratio by volume). Salt solutions containing 200 mM, 200 mM and 100 mM NaCl were applied to five‐week‐old tomato seedlings on the 1st, 8th and 15th day, respectively. Phenotyping was performed 7 days after the plants were irrigated with the third salt solution. To induce salt stress in *Arabidopsis*, seedlings were grown on an MS medium for 9 days with no salt, 100 mM or 125 mM NaCl. In vertical culture, seedlings were grown on an MS medium containing no salt, 50 mM, 100 mM or 125 mM NaCl. Transgenic lines were also grown in plastic pots (7×7×7 cm) filled with compound soil, with four replicates per line and five individuals per replicate. A solution of 200 mM NaCl was applied twice (once a week) to two‐week‐old plants.

### Other methods

Details of the methods for physiological measurements and histochemical staining, yeast two‐hybrid (Y2H) assay, subcellular localization and bimolecular fluorescence complementation (BiFC) assays, luciferase transient expression assay in tobacco leaves, Co‐immunoprecipitation (Co‐IP) and Western blotting, RNA isolation and quantitative RT‐PCR (qRT‐PCR) analysis with specific primers (Table S3) are available in Appendix S1 at PBJ online.

## Supporting information


**Figure S1** Amino acid sequence alignment of SlZF3 and homologous zinc‐finger proteins from *Arabidopsis* and tomato.

**Figure S2** Subcellular localization of SlZF3 and related proteins.

**Figure S3** Interaction between VTC1 and CSN5B detected by BiFC.

**Figure S4** Relative expression level of *SlZF3* in different transgenic lines of tomato and *Arabidopsis*.

**Figure S5** Overexpression of *SlZF3* enhances the salt tolerance of *Arabidopsis* in soil.

**Figure S6** Expression of key genes involved in AsA biosynthesis and metabolism.

**Table S1** Primers used for cloning and vector construction.
**Table S2** Genes identified using the yeast two‐hybrid screen.
**Table S3** Primers used for qRT‐PCR analysis.

**Appendix S1** Supporting methods.
