# Supplementary figures and images for: The C2H2 zinc‐finger protein SlZF3 regulates AsA synthesis and salt tolerance by interacting with CSN5B
Source: Plant Biotechnol J. 2017 Dec 28;16(6):1201–13. doi: 10.1111/pbi.12863 (PMC5978872; doi:10.1111/pbi.12863)

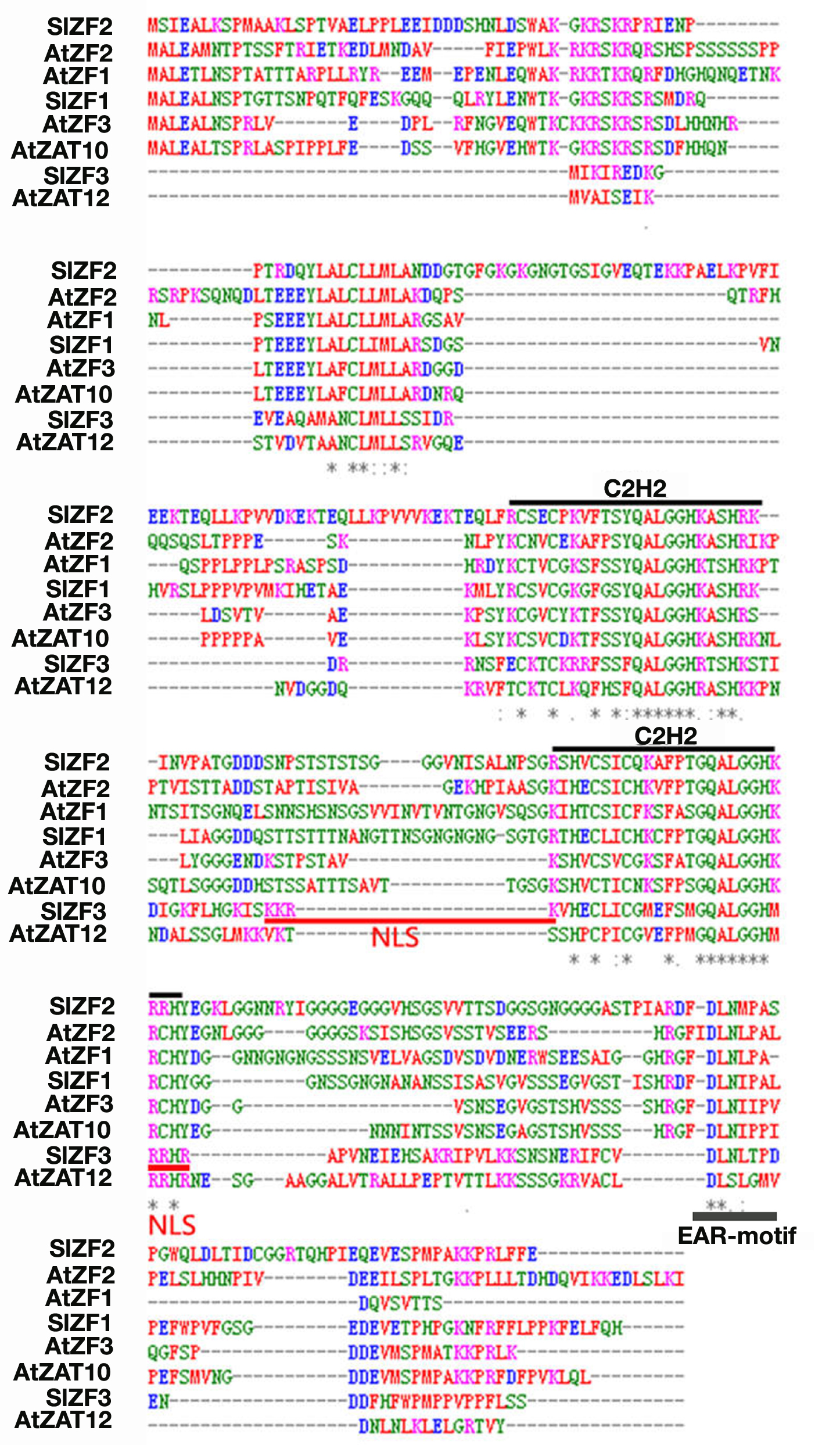

Supplement: Supplementary file 1 — Figure S1 Amino acid sequence alignment of SlZF3 and homologous zinc‐finger proteins from Arabidopsis and tomato. [file PBI-16-1201-s006.jpg]

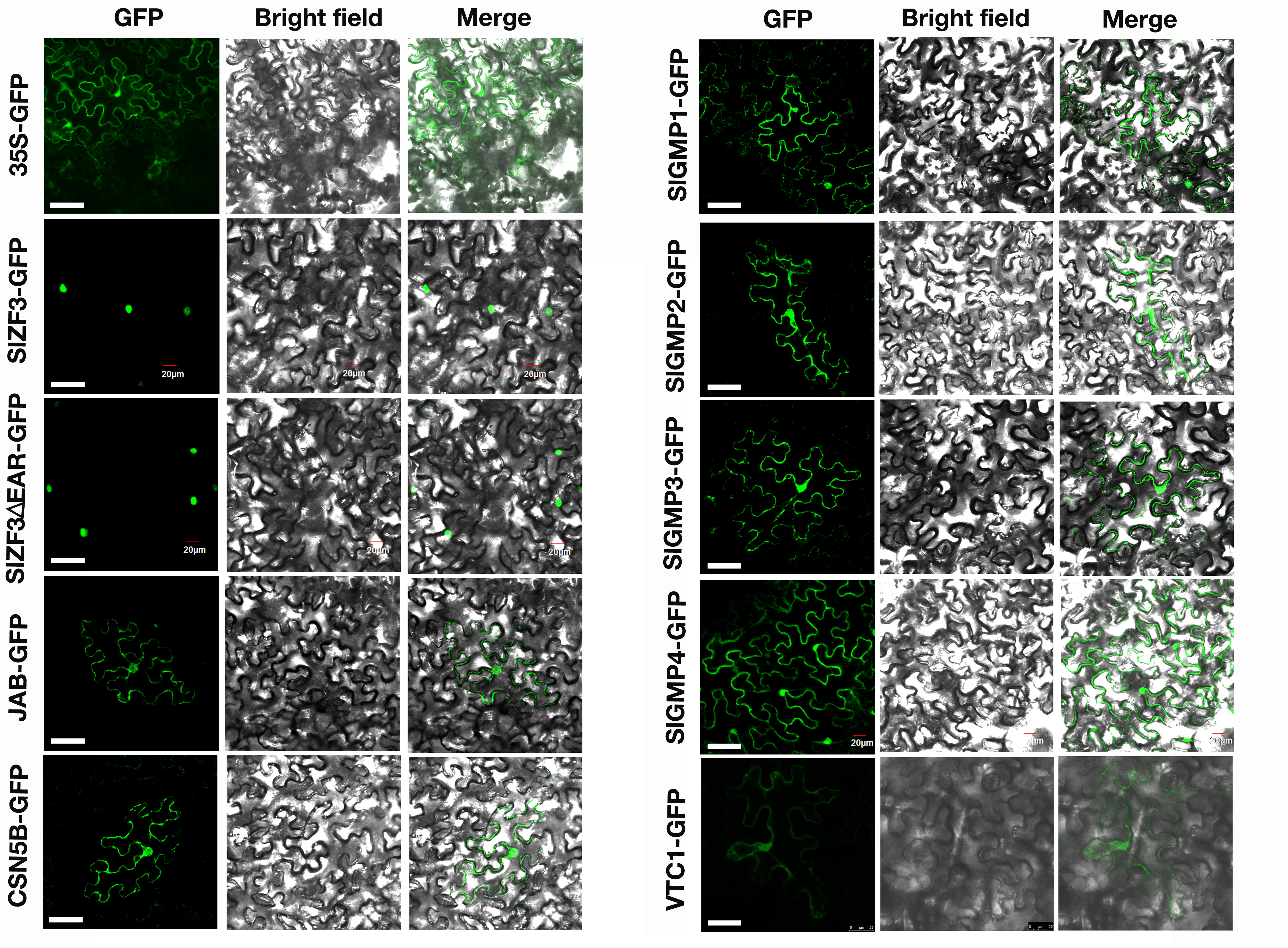

Supplement: Supplementary file 2 — Figure S2 Subcellular localization of SlZF3 and related proteins. [file PBI-16-1201-s005.jpg]

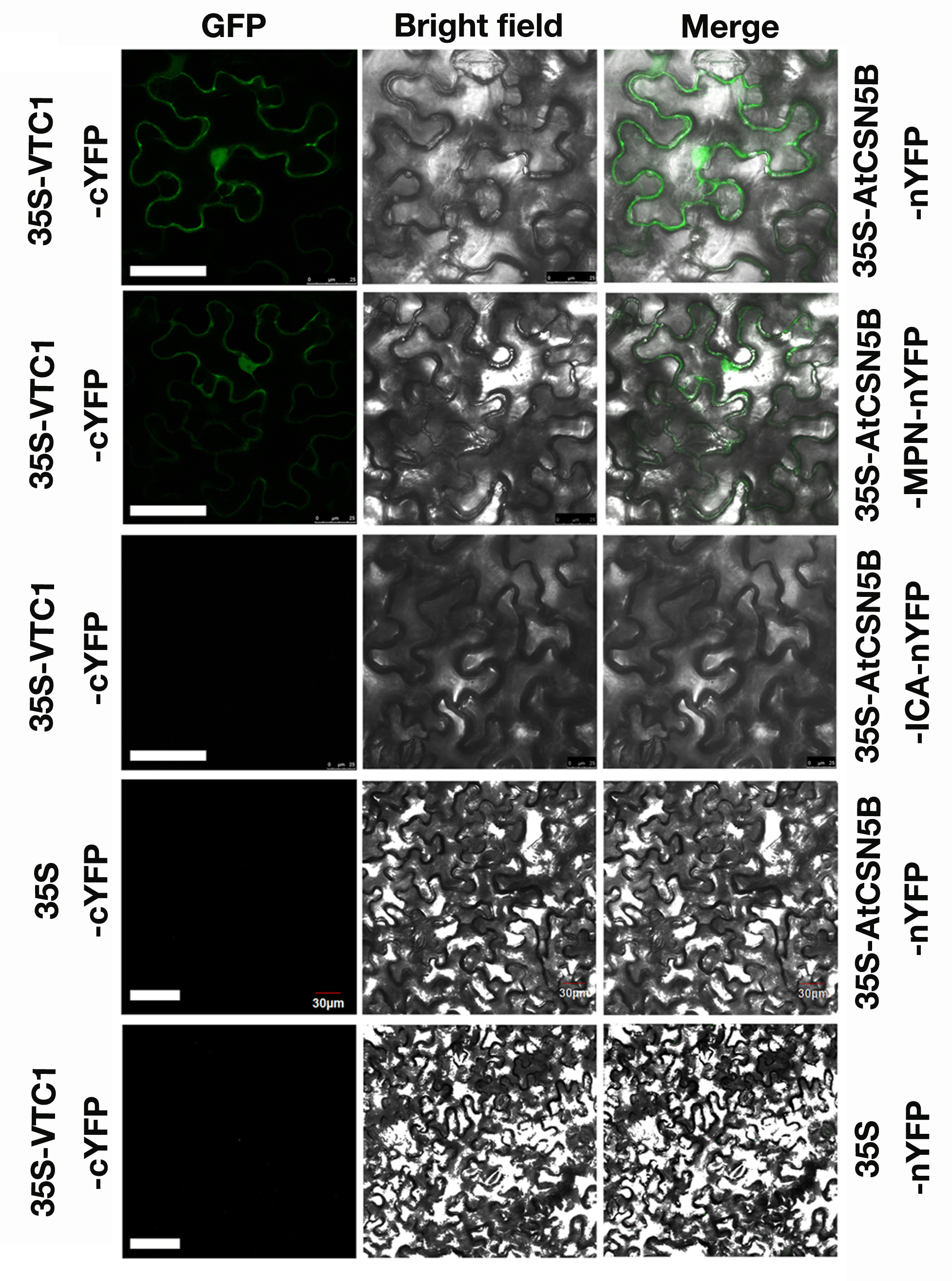

Supplement: Supplementary file 3 — Figure S3 Interaction between VTC1 and CSN5B detected by BiFC. [file PBI-16-1201-s004.tif]

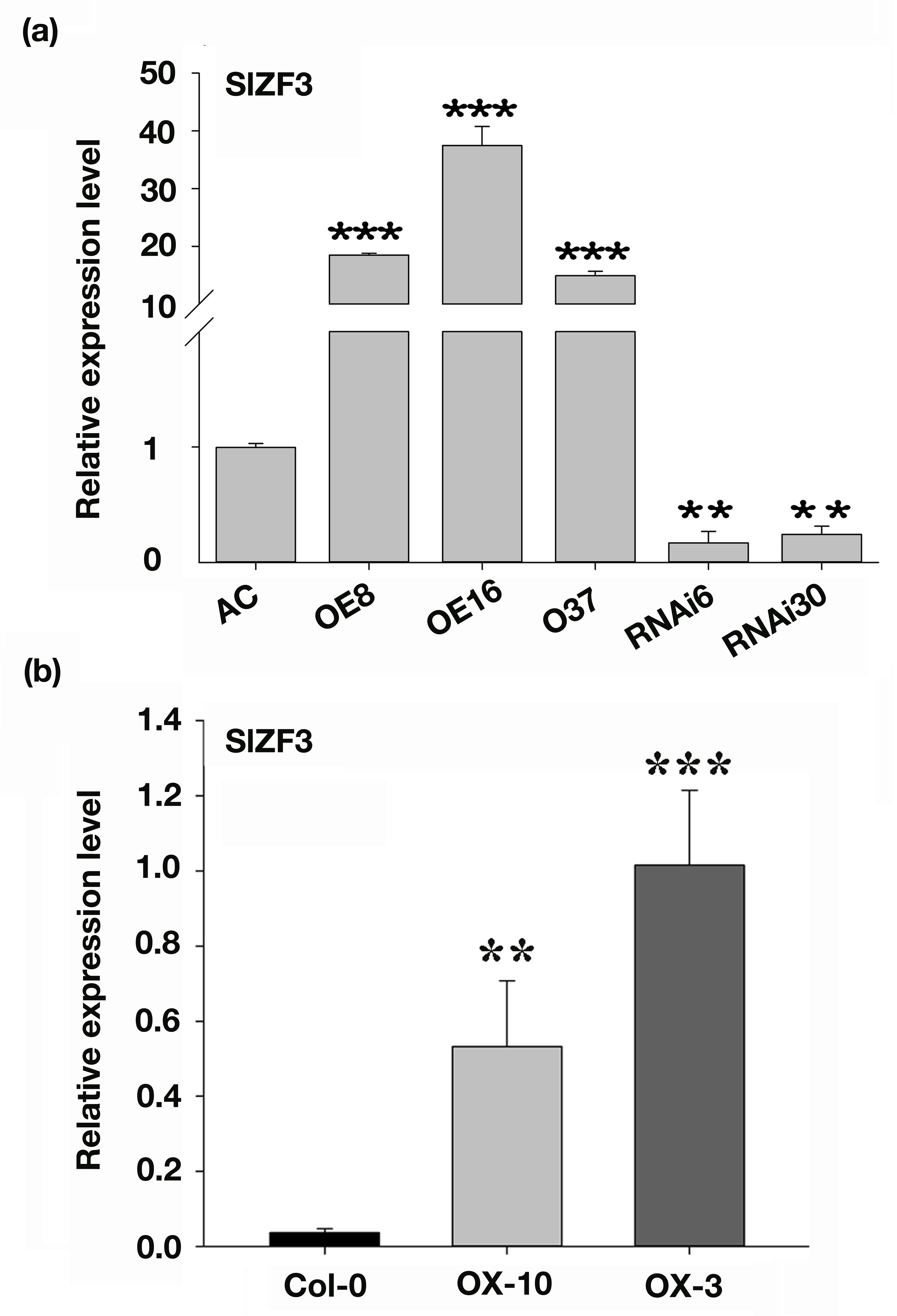

Supplement: Supplementary file 4 — Figure S4 Relative expression level of SlZF3 in different transgenic lines of tomato and Arabidopsis. [file PBI-16-1201-s008.jpg]

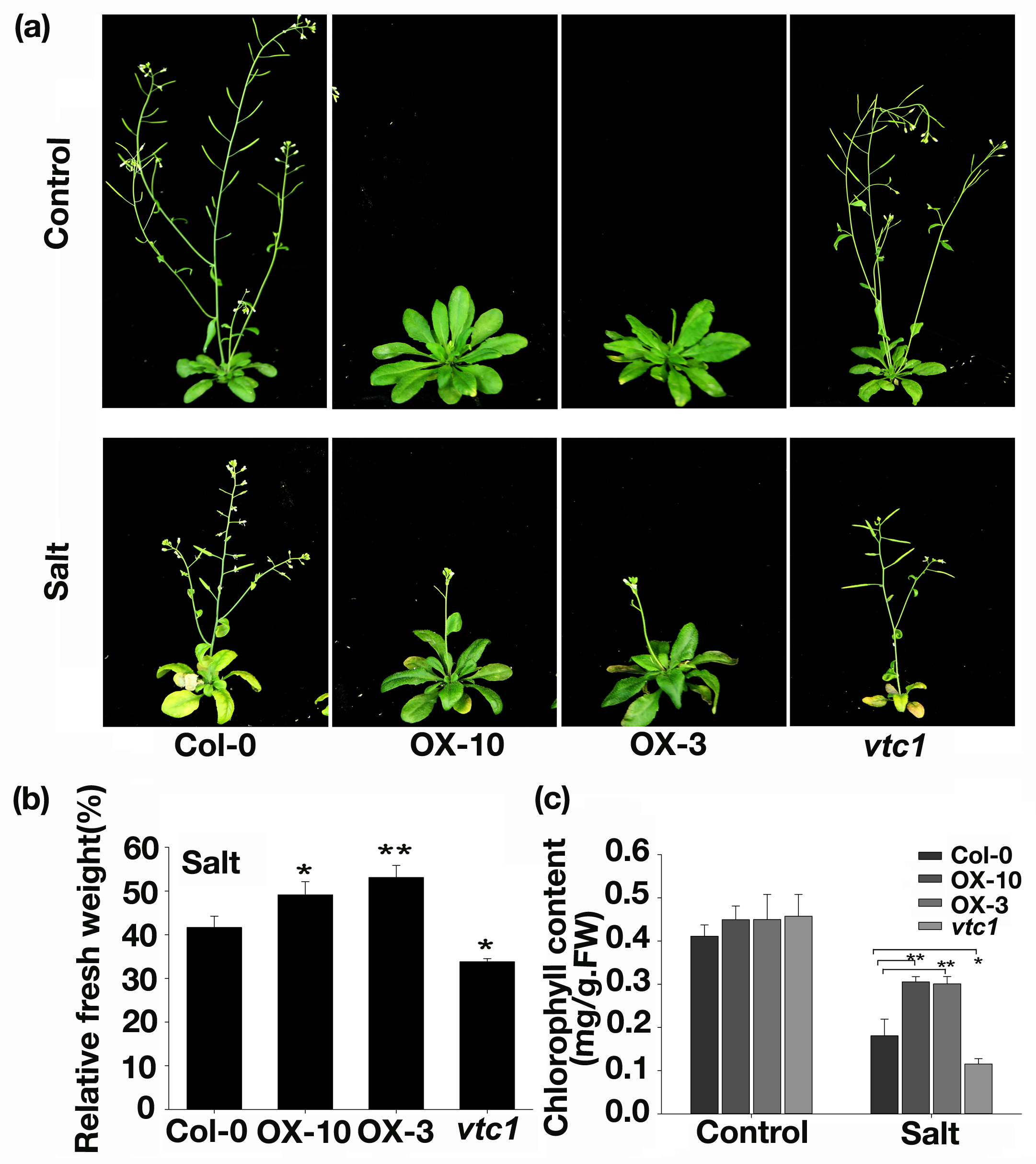

Supplement: Supplementary file 5 — Figure S5 Overexpression of SlZF3 enhances the salt tolerance of Arabidopsis in soil. [file PBI-16-1201-s003.jpg]
