# Supplementary material for: The C2H2 zinc‐finger protein SlZF3 regulates AsA synthesis and salt tolerance by interacting with CSN5B
Source: Plant Biotechnol J. 2017 Dec 28;16(6):1201–13. doi: 10.1111/pbi.12863 (PMC5978872; doi:10.1111/pbi.12863)
Supplement: Supplementary file 7 — Table S1 Primers used for cloning and vector construction. Table S2 Genes identified using the yeast two‐hybrid screen. Table S3 Primers used for qRT‐PCR analysis. [file PBI-16-1201-s001.docx]

**Supporting tables**

Table S1. Primers for cloning and vector construction.

| Name | Forward (5’→3’) | Reverse (5’→3’) | Usage |
| --- | --- | --- | --- |
| BD-SlZF3 | TCCCCCGGGATGATAAAAATTAGAGAAG | CCGCTCGAGCTAAGAGGATAAGAATGGAGGTAC | Y2H |
| AD-AtCSN5B | CCGGAATTCATGGAGGGTTCGTCGTCGACGATAG | CGAGCTCTCAATATGTAATCATAGGGTCTGG | Y2H |
| TAZF3-1 | GAATTCATGATAAAAATTAGAGAAG |  | Bridge PCR for EAR deletion |
| TAZF3-2 | CTCATCCACGCAAAATATTCTC |  | Bridge PCR for EAR deletion |
| TAZF3-3 | GCGTGGATGAGAATGATGAC |  | Bridge PCR for EAR deletion |
| TAZF3-4 | CTGCAGGCTAAGAGGATAAGAATGGAGGTAC |  | Bridge PCR for EAR deletion |
| BD-SlZF3△EAR | TCCCCCGGGATGATAAAAATTAGAGAAG | CCGCTCGAGCTAAGAGGATAAGAATGGAGGTAC | Y2H |
| AD-SlCSN5B | TCCCCCGGGATGGACGCTCTGAATTCTTACG | CCGCTCGAGTCAGGTTTCGACCATCGGCTCTGG | Y2H |
| CE-SlZF3 | TTGGCGCGCCATGATAAAAATTAGAGAAGAC | TCCCCCGGGAGAGGATAAGAATGGAGGTAC | BIFC |
| NE-AtCSN5B | TTGGCGCGCCATGGAGGGTTCGTCGTCGACGATAG | TCCCCCGGGATATGTAATCATAGGGTCTGG | BIFC |
| CE-SlZF3△EAR | GGCGCGCCATGATAAAAATTAGAGAAG | TCCCCCGGGAGAGGATAAGAATGGAGGTAC | BIFC |
| NE-SlCSN5B | GGCGCGCCATGGACGCTCTGAATTCTTACG | TCCCCCGGGGGTTTCGACCATCGGCTCTGG | BIFC |
| AtCSN5B-NLuc | cgagctcggtacccgggatccATGGAAGGTTCCTCGTCAGCC | CGCGTACGAGATCTGgtcgacCGATGTAATCATGGGCTCTGG | LUC |
| At1VTC-CLuc | tacgcgtcccggggcggtaccATGAAGGCACTCATTCTTGTTG | ACGAAAGCTCTGCAGgtcgacTCACATCACTATCTCTGGC | LUC |
| ZAT12-Flag | GATATAACATTACGCATGGTTGCGATATCGGAG | GTAATCATAGGGAAGATAAACTGTTCTTCCAAGCTCC | Co-IP/Western blot |
| SlZF3-Flag | GATATAACATTACGCATGATAAAAATTAGAGAAG | GTAATCATAGGGAAGAGAGGATAAGAATGGAGGTAC | Co-IP/Western blot |
| AtCSN5B-myc | GATATAACATTACGCATGGAAGGTTCCTCGTCAGCC | CTCCATATAGGGAAGCGATGTAATCATGGGCTCTGG | Co-IP/Western blot |
| AtVTC1-GFP | GATATAACATTACGCATGAAGGCACTCATTCTTGTTG | CACCATATAGGGAAGCATCACTATCTCTGGCTTCAAG | Co-IP/Western blot |
| ZF3-1302 | ACGGGGGACTCTTGAccatggTAATGATAAAAATTAGAGAAG | AAGTTCTTCTCCTTTACTAGTCTAAGAGGATAAGAATGGAGGTAC | Subcellular localization |
| SlGMP1-1302 | ACGGGGGACTCTTGAccatggTAATGGGAAGTTCTGAGGAGAAAG | AAGTTCTTCTCCTTTACTAGTAAGTAAGATCTCTTCTTGGAC | Subcellular localization |
| SlGMP2-1302 | ACGGGGGACTCTTGAccatggTAATGAAGGCACTTATTCTTGTTG | AAGTTCTTCTCCTTTACTAGTCATCACTATTTCTGGTTTTAG | Subcellular localization |
| SlGMP3-1302 | ACGGGGGACTCTTGAccatggTAATGAAGGCACTTATCCTTGTTG | AAGTTCTTCTCCTTTACTAGTCATCACGATTTCAGGTTTCAA | Subcellular localization |
| SlGMP4-1302 | ACGGGGGACTCTTGAccatggTAATGAAGGCGCTCATCCTTGTAG | AAGTTCTTCTCCTTTACTAGTCATGACAATCTCTGGCTCCAG | Subcellular localization |
| JAB-1302 | ACGGGGGACTCTTGAccatggTAATGGACGCTCTGAATTCTTAC | AAGTTCTTCTCCTTTACTAGTTCAGGTTTCGACCATCGGCTC | Subcellular localization |
| AtCSN5B-1302 | ACGGGGGACTCTTGAccatggTAATGGAAGGTTCCTCGTCAGCC | AAGTTCTTCTCCTTTACTAGTTCACGATGTAATCATGGGCTC | Subcellular localization |
| VTC1-1302 | ACGGGGGACTCTTGAccatggTAATGAAGGCACTCATTCTTGTTG | AAGTTCTTCTCCTTTACTAGTCATCACTATCTCTGGCTTCAAG | Subcellular localization |
| ZF3-CE(M) | GAGAACACGGGGGACTCTAGAATGATAAAAATTAGAGAAGAC | CCCGGGAGCGGTACCCTCGAGAGAGGATAAGAATGGAGGTAC | BIFC |
| VTC1-CE(M) | GAGAACACGGGGGACTCTAGAATGAAGGCACTCATTCTTGTTG | CCCGGGAGCGGTACCCTCGAGCATCACTATCTCTGGCTTCAAG | BIFC |
| SlGMP1-CE(M) | GAGAACACGGGGGACTCTAGAATGGGAAGTTCTGAGGAGAAAG | CCCGGGAGCGGTACCCTCGAGAAGTAAGATCTCTTCTTGGAC | BIFC |
| SlGMP2-CE(M) | GAGAACACGGGGGACTCTAGAATGAAGGCACTTATTCTTGTTG | CCCGGGAGCGGTACCCTCGAGCATCACTATTTCTGGTTTTAG | BIFC |
| SlGMP3-CE(M) | GAGAACACGGGGGACTCTAGAATGAAGGCACTTATCCTTGTTG | CCCGGGAGCGGTACCCTCGAGCATCACGATTTCAGGTTTCAA | BIFC |
| SlGMP4-CE(M) | GAGAACACGGGGGACTCTAGAATGAAGGCGCTCATCCTTGTAG | CCCGGGAGCGGTACCCTCGAGCATGACAATCTCTGGCTCCAG | BIFC |
| JAB-NE(R)173 | CCCAGGCCTACTAGTGGATCCATGGACGCTCTGAATTCTTAC | CTCCTACCCGGGAGCGGTACCTCAGGTTTCGACCATCGGCTC | BIFC |
| JAB-MPN(R)NE173 | CCCAGGCCTACTAGTGGATCCATGGACGCTCTGAATTCTTAC | CTCCTACCCGGGAGCGGTACCTCAAAGAAAGGGCTCCTGATA | BIFC |
| JAB-ICA(R)NE173 | CCCAGGCCTACTAGTGGATCCGCAGTTGTTATTGATCCAAC | CTCCTACCCGGGAGCGGTACCTCAGGTTTCGACCATCGGCTC | BIFC |
| AtCSN5B-NE(R)173 | CCCAGGCCTACTAGTGGATCCATGGAAGGTTCCTCGTCAGCC | CTCCTACCCGGGAGCGGTACCTCACGATGTAATCATGGGCTC | BIFC |
| AtCSN5B-MPN(R)NE173 | CCCAGGCCTACTAGTGGATCCATGGAAGGTTCCTCGTCAGCC | CTCCTACCCGGGAGCGGTACCTCACTAAGAATGGCTCCTCATAC | BIFC |
| AtCSN5B-ICA(R)NE173 | CCCAGGCCTACTAGTGGATCCCTGTTGTTATTGATCCAAC | CTCCTACCCGGGAGCGGTACCTCACGATGTAATCATGGGCTC | BIFC |
| VTC1-NE(R)173 | CCCAGGCCTACTAGTGGATCCATGAAGGCACTCATTCTTGTTG | CTCCTACCCGGGAGCGGTACCTCACATCACTATCTCTGGCTTCAAG | BIFC |
| SlGMP3-NE(R)173 | CCCAGGCCTACTAGTGGATCCATGAAGGCACTCATTCTTGTTG | CTCCTACCCGGGAGCGGTACCTCACATCACGATTTCAGGTTTCAA | BIFC |

Table S2. Genes identified in the yeast two hybridization screening

| Counts | TAIR ID | Function description |
| --- | --- | --- |
| 13 | AT4G32530 | ATPase, F0/V0 complex, subunit C protein mRNA |
| **10** | **AT1G71230** | **COP9 signalosome complex subunit 5b mRNA** |
| 10 | AT3G47650 | DnaJ/Hsp40 cysteine-rich domain-containing protein mRNA |
| 10 | AT3G53430 | mRNA for 60S ribosomal Protein L12 -like |
| 7 | AT2G04700 | putative ferredoxin-thioredoxin reductase mRNA |
| 5 | AT1G47128 | cysteine proteinase RD21a mRNA |
| 2 | AT3G54826 | Zim17-type zinc finger protein mRNA |
| 2 | AT1G09070 | protein SRC2 mRNA |
| 1 | AT3G55380 | UbcAT3 mRNA for ubiquitin conjugating enzyme E2 |
| 1 | AT5G62300 | 40S ribosomal protein S20-1 mRNA |
| 1 | AT4G33680 | LL-diaminopimelate aminotransferase mRNA |
| 1 | AT1G14150 | photosynthetic NDH subcomplex L2 mRNA |
| 1 | AT4G36010 | pathogenesis-related thaumatin family protein mRNA |
| 31 |  | unknown proteins |

Table S3. Primers used for qRT-PCR analysis

| Name | Forward (5’→3’) | Reverse (5’→3’) |
| --- | --- | --- |
| QVTC1 | CAAAACCGGTCCAACACCAGA | CTGAGCTGGGAAATGGCTGTA |
| QVTC2 | GGTGTACCAATAGCATCTCA | CACCGGCGAGAAAGAGGAGA |
| QVTC4 | GGATGAGTGGTTCGTGTGCA | CTCAGCGAATAACTCCTTGA |
| QVTC5 | AATGTGAGTCCGATTGAGTA | AGTAAGCCTGAAAGTGAAGA |
| QGME | CGATGAGTGTGTTGAAGG | AGATTGTTGTCTGAGTTACG |
| QGLDH | GCAGATTGGTGGTATTATTC | GACCTCAGCAACAACTCC |
| QMIOX4 | ATGAGGTTGTGGATGAGAG | TGTCAGAGAGGTTAATCAGA |
| QAPX3 | GGAAAGCTAAGAATTTGAACTGA | GAGACAGAGAAGAGAGAAGA |
| QChIDHAR | CTAGTCTAGCTAAGGGACAA | GTTGACTGACACGTGGCGAAA |
| QCytDHAR | GCTCAGAGGCTTCCACACACCA | ATCCCACGGAGTCGAAGAA |
| QGalDH | CTCTGACAGAAAGCTCATGA | GAGAGACGCGAGAAGACAACA |
| QAtACTIN | AACAGCAGAACGGGAAATTGTGAGA | TGGAGGAGCTGGTTTTCGAGGT |
| QSlGMP1 | CAGGTAGCATTATCGGTTGG | TTGATCTCCTTGTGGGGTAA |
| QSlGMP2 | GTGTAGTTTTGCCCCACAAG | AGGAGAACTGGAAACCAACC |
| QSlGMP3 | AGAAGCGACTGGGAGAGTTG | GAATCCGGTCCAAAACAGAA |
| QSlGMP4 | AGACAGAGCCTTTAGGCACAGC | GCCGTGGGACTTATGAAACAA |
| QSlGME1 | TTCCTGTCCAACACATTCCT | CTTTCTCGATCTGCTCCTTG |
| QSlGME2 | ATTCGAGATGTGGGGAGATG | GGAAGGTTCTTGCCATCAAA |
| QSlGGP | GAAATCTGGTCTGTTCCTCTGTGA | TTCACACACCAACTCCACATTACA |
| QSlGP1 | TTCACACACCAACTCCACATTACA | TGTCCGCTTTCCATCTCCTAT |
| QSlGalDH | CTTCTTACTGAGGCTGGTGGTC | AACCTCTTTAACAGACTTCATCCC |
| QSlGLDH | ATTGAGGTTCCCAAGGACATAG | ATGTTATTAGATAGGATGCGGTTT |
| QSlMDHAR | GGTGATGTTGCCACTTTTCCTTT | CGACAGACTTCCCTTGCTCACT |
| QSlDHAR | CCTACCTTCGTCTCATTTCCG | TGAACAAACATTCTGCCCATT |
| QSlcAPX | ACGATGATATTGTGACACTCTTCCA | AAGCGATGAAACCACAAAAACA |
| QSlMIOX | ACTACTCTTCCTTCTGCTGCTTTA | AATGTTGAGCCACTTCATGTTCT |
| QSlACTIN | GAAATAGCATAAGATGGCAGACG | ATACCCACCATCACACCAGTAT |
| QSlZF3 | GAAGAAATCGAATAGCAACGAGAG | GAATGGAGGTACAGGTGGCATAG |
| QJAB | CGCAACAAACATGGGAGTTA | ATCCGACGCAGAGTTTTCC |
| QAtCSN5B | AGGTTCCTCGTCAGCCATC | AGGCTGAATCGGTTGGTTC |
