# Supplementary material for: The C2H2 zinc‐finger protein SlZF3 regulates AsA synthesis and salt tolerance by interacting with CSN5B
Source: Plant Biotechnol J. 2017 Dec 28;16(6):1201–13. doi: 10.1111/pbi.12863 (PMC5978872; doi:10.1111/pbi.12863)
Supplement: Supplementary file 8 — Appendix S1 Supporting methods. [file PBI-16-1201-s007.docx]

**Supporting methods:**

**Physiological measurements and histochemical staining**

AsA content was measured as described by Stevens *et al.* (2006). Rosette leaves from salt-treated *Arabidopsis* seedlings or the 3rd leaf from the top of two-month-old tomato plants were collected, frozen, and ground into fine powder using liquid nitrogen. Approximate 200 mg of ground tissue was added to 0.6 mL of ice-cold 6% trichloroacetic acid (TCA) in a 2 mL Eppendorf tube. The samples were vortexed for 10 s and incubated on ice for 15 min to quench the metabolism. The homogenate was centrifuged at 25, 000 × g for 10 min at 4 °C, and the supernatant was transferred to a new Eppendorf tube for subsequent analysis. The AsA standard (Sigma-Aldrich, USA, A7631) was prepared fresh on the day of the analysis. The absorbance was recorded at 550 nm using an Infinite M200 (Tecan, Switzerland, Infinite M200 Pro).

The measurement of H_2_O_2_ levels was performed as described by Huang et al. (2013). Samples were collected as described for AsA analysis. The H_2_O_2_ levels were quantified using the appropriate detection kits (Jiancheng Bioengineering Institute, Nanjing, China) according to the manufacturer’s instructions.

The measurement of chlorophyll and MDA content were performed as recommended by Zhang *et al*. (2012). The samples were prepared as described for quantitation of AsA. Chlorophyll and MDA were extracted from approximately 100 mg of ground tissue. The absorbance of chlorophyll was recorded at 664 and 647 nm using an Infinite M200 spectrophotometer (Tecan). The MDA content was quantified using spectrophotometry by measuring the absorbance at 532 and 600 nm.

The tomato leaves—the 3rd leaf from the top—were infiltrated with 30 mL of DAB (1 mg mL^–1^, pH 3.8) and NBT (0.5 mg mL^–1^ NBT dissolved in 25 mM phosphate buffer saline (PBS), pH 7.8), respectively, and placed in the dark overnight as recommended by Ma *et al*. (2013).

The GUS staining of tomato seedlings was performed as recommended by Dobisova *et al*. (2017). Stained tissues were imaged using differential interference contrast (DIC) microscopy as described by Beeckman and Engler (1994).

**Yeast two-hybrid (Y2H) assay**

An *Arabidopsis* cDNA expression library was constructed using the Clontech Make Your Own "Mate & Plate™" Library System (Clontech, Mountain View, CA, USA) using mRNA from various tissues of *Arabidopsis* plants, namely roots, stems, immature and mature leaves, flowers, immature and mature siliques. *SlZF3* or *SlZF3* without the sequence for the EAR motif (i.e., *SlZF3ΔEAR*) was cloned into pGBKT7 between the *EcoR*I and *Pst*I restriction sites. The coding sequences (CDS) of *CSN5B* and *JAB* were separately cloned into pGADT7 between *EcoR*I and *Sac*I using specific oligonucleotides (Table S2). To test the transactivation activity of SlZF3, the pGBKT7-SlZF3 + pGADT7-EV (Empty vector), pGBKT7-Lam + pGADT7-RecT (negative control) and pGBKT7-53 + pGADT7-RecT (positive control) were co-transformed into AH109 cells, respectively. Yeast clones were grown on SD/-Trp/X-α-Gal or SD/-Ade/-His/-Trp/X-α-Gal medium for 3-4 days at 30 °C. The cDNA clones in pGADT7 in yeast strain Y187 and pGBKT7-SlZF3 or pGBKT7-SlZF3ΔEAR in AH109 were mated for 20 to 24 h. Positive clones were tested for the presence of appropriate plasmids using clony-PCR. The amplified fragments longer than 500 bp were selected for sequencing.

To test for interactions between SlZF3 and both CSN5B and JAB, yeast AH109 cells were co-transformed with either pGADT7-AtCSN5 or pGADT7-SlCSN5 and either pGBKT7-SlZF3 or pGBKT7-SlZF3ΔEAR. Yeast cells were grown on SD/-Trp/-Leu medium without X-α-Gal and SD/-Ade/-His/-Trp/-Leu medium supplemented with X-α-Gal at 30 °C for 3 to 4 d. As described above, pGBKT7-53 + pGADT7-RecT, and pGBKT7-Lam + pGADT7-RecT were used as positive and negative controls, respectively.

**Subcellular localization and** **bimolecular fluorescence complementation (BiFC) assays**

*Arabidopsis* mesophyll cell protoplasts were prepared from the rosette leaves of four-week-old plants, and subcellular localization analysis was performed as described by Yoo *et al*. (2007). The BiFC analysis was carried out as described by Walter *et al*. (2004). The coding sequences of SlZF3 without termination codons were cloned into pMDC83 (Curtis and Grossniklaus, 2003), using specific oligonucleotides (Table S2) and the Gateway LR reaction (Invitrogen). For BiFC analysis, the *SlZF3* CDS or *SlZF3ΔEAR* without a termination codon was cloned into pUC-SPYCE-35S-cYFP and the CDS of *CSN5B* or *JAB* was cloned into pUC-SPYNE-35S-nYFP, respectively. *Asc*I, *Sma*I and specific oligonucleotides (Table S2) were employed in the cloning process. The plasmids 35S-cYFP with 35S-nYFP, 35S-SlZF3-cYFP with 35S-nYFP, 35S-cYFP with 35S-JAB-nYFP, and 35S-SlZF3ΔEAR-cYFP with 35S-nYFP were used as the negative controls. The rice GHD7 (GenBank Accession No. EU286800) was fused to cyan fluorescent protein (CFP) and used as a marker for nuclear-localization. After transformation and an overnight incubation, the YFP signals were detected by confocal laser scanning microscopy.

Leaves from tobacco (*Nicotiana benthamiana*) were also used for subcellular localization and protein-protein interaction analyses. The coding sequences without the termination codon from *SlZF3*, *SlZF3* without the EAR-motif (*SlZF3ΔEAR*), *JAB*, *CSN5B*, *SlGMP1*, *SlGMP2*, *SlGMP3*, *SlGMP4*, and *VTC1* were cloned into pCAMBIA1302-GFP (Chen *et al*., 2015) *via* recombination as recommended in the ClonExpress® Entry One Step Cloning Kit manual (Vazyme Biotech, China, C112-02). For BiFC analysis, coding sequences from *SlZF3*, *SlGMP1*, *SlGMP2*, *SlGMP3* and *SlGMP4* were fused in-frame to the C-terminal end of YFP in pSPYCE (M) using the *Xba*I and *Xho*I sites. The coding sequences of *JAB*, *JAB-MPN* (the MPN domain of JAB), *JAB-ICA* (the ICA domain of JAB), *CSN5B*, *CSN5B-MPN* (the MPN domain of CSN5B), *CSN5B-ICA* (the ICA domain of CSN5B), *SlGMP3* without the stop codons were fused to the N-terminus of YFP in pSPYNE(R)173 using the *Bam*HI and *Kpn*I sites (Waadt *et al*., 2008). All of the primers used to construct these plasmids are listed in Table S2. The resulting vectors were used to transform *Agrobacterium* *tumefaciens* GV3101. Overnight cultures of Agrobacteria harboring the above constructs were suspended in infiltration medium (10 mM MES, 10 mM MgCl_2_, 150 µM acetosyringone, pH 6.0) to an OD_600_ of 0.4 to 0.5. The cultures were spot-infiltrated into 5- to 6-week-old tobacco leaves. YFP signals were detected by confocal laser scanning microscopy 48 h after infiltration.

**Luciferase transient expression assay in *Nicotiana benthamiana* leaves**

The luciferase transient expression assay was performed as described by Chen *et al*. (2008) with minor modifications. The coding sequences of *CSN5B* and *VTC1* without the stop codon were cloned into the pCAMBIA-NLuc and pCAMBIA-CLuc between *Kpn*I and *Sal*I. The resulting vectors were named CSN5B-NLuc (pCAMBIA-NLuc, 35S promoter) and VTC1-CLuc (pCAMBIA-CLuc, 35S promoter), respectively. The coding sequences for *CSN5B* and *AtZAT12* without termination codons were separately cloned into the pENT4 vector (http//www.invitrogen.com/support) between the two *Eco*RI restriction sites and were subsequently cloned into the pK2GW7 (Karimi *et al*., 2002) using specific oligonucleotides (Table S2) and Gateway LR reactions (Invitrogen). The resulting vectors were named SlZF3-FLAG (35S::SlZF3-FLAG) and ZAT12-FLAG (35S::ZAT12-FLAG).

The Agrobacteria infiltration was carried out as above. The competition assays were performed with cultures of CSN5B-NLuc, VTC1-CLuc and either SlZF3-Flag or ZAT12-Flag at ratios of 1:1:1, 1:1:10 or 1:10:1, respectively. Bioluminescence was imaged using a CCD camera (Lumazone Pylon 2048 B, 16bits, Roper Scientific) 72 h after infiltration. An exposure time of 10 min was used for all of the images that were collected.

**Co-immunoprecipitation (Co-IP) and western blotting**

Co-immunoprecipitation and western blotting were performed as described previously by Huang *et al*. (2013) with minor modifications. The CDSs without stop codons from *SlZF3*, *SlZF3ΔEAR*, *CSN5B* and *VTC1* were cloned in frame and upstream of the FLAG epitope (pH7LIC4.1), myc epitope (pH7LIC10.1) and GFP (pH7LIC6.0) using specific oligonucleotides (Table S2) and the ClonExpress® Entry One Step Cloning Kit (Vazyme Biotech), respectively. The above vectors were kindly provided by Dr. Feng Li. *N. benthamiana* leaves were infiltrated with *Agrobacterium tumefaciens* strain GV3101 harboring plasmids that encode the SlZF3-FLAG, SlZF3ΔEAR-FLAG, CSN5B-myc, and VTC1-GFP fusion proteins at an OD_600_ of 0.4 and also including 100 μM MG132 for the Co-IP analysis. Leaf tissue was harvested 2 d after infiltration and ground to a fine powder in liquid nitrogen. Samples were suspended in ice-cold extraction buffer (50 mM Tris-HCl, pH 7.5, 150 mM NaCl, 5 mM EDTA, 2 mM DTT, 10% glycerol, 1 mM PMSF and 10 μL mL^-1^ plant protease inhibitor cocktail). After 30 min of extraction on ice, the samples were centrifuged at 15000 × g for 30 min. For co-immunoprecipitation, one-tenth of each supernatants (v: v) was saved as the input material. The rest of the supernatant was incubated with anti-FLAG agarose beads (MBL, Japan, M185-10) for 2 h at 4°C. The agarose beads were washed four times with the extraction buffer. The immunoprecipitates were eluted with 1× SDS sample buffer (50 mM Tris-HCl, pH 6.8, 2% SDS, 10% glycerol, 0.1% bromophenol blue, 1% 2-mercaptoethanol) and incubated at 100 °C for 5 min to release the immunoprecipitated complex. After centrifuging at 500 × g for 15 s, supernatants (200 μL) were mixed with 50 μL 5 × SDS sample buffer and incubated at 100 °C for 5 min. The samples were analyzed using a 12% SDS gel and immunoblotting with the appropriate primary antibody (i.e. anti-FLAG, anti-myc or anti-GFP).

**RNA isolation and quantitative RT-PCR (qRT-PCR)**

Total RNA was extracted using the TRIzol reagent (Invitrogen) according to the manufacturer's instructions. Amplification was conducted in 96-well optical reaction plates on a LightCycler 480 real-time PCR machine (Roche-Diagnostics, Basel, Switzerland), with the following program: 95 °C for 30 s, 40 cycles of 95 °C for 5 s, and 60 °C for 25 s. Each reaction contained 10 μL SYBR Premix Ex Taq (Takara, Kyoto, Japan), 1.0 μL cDNA sample, and 0.8 μL of 10 μM gene-specific primers (Table S3) in a final volume of 20 μL. Relative gene expression was calculated using the comparative C_T_ method (Schmittgen and Livak, 2008). The tomato actin gene (GenBank Accession No. BT012695) was used as the internal control. Statistical significance was determined using the Student’s t test.

Reference

Beeckman, T. and Engler, G. (1994) An easy technique for the clearing of histochemically stained plant tissue. *Plant Mol. Biol. Rep.* **12**, 37-42.

Chen, H., Zou, Y., Shang, Y., Lin, H., Wang, Y., Cai, R., Tang, X. and Zhou, J.M. (2008) Firefly luciferase complementation imaging assay for protein-protein interactions in plants. *Plant Physiol*. **146**, 368-376.

Chen, J., Yang, L., Gu, J., Bai, X., Ren, Y., Fan, T., Han, Y., Jiang, L., Xiao, F. and Liu, Y. (2015) MAN3 gene regulates cadmium tolerance through the glutathione-dependent pathway in *Arabidopsi*s thaliana. *New Phytol*. **205**, 570.

Curtis, M.D. and Grossniklaus, U. (2003). A gateway cloning vector set for high-throughput functional analysis of genes in planta. *Plant Physiol*. 133.2: 462-469.

Dobisova, T., Hrdinova, V., Cuesta, C., Michlickova, S., Urbankova, I., Hejatkova, R., Zadnikova, P., Pernisova, M., Benkova, E., Hejatko, J. (2017) Light controls cytokinin signaling via transcriptional regulation of constitutively active sensor histidine kinase CKI1. *Plant Physiol*. **174**,387-404.

Huang, X.S., Wang, W., Zhang, Q. and Liu, J.H. (2013) A basic helix-loop-lelix transcription factor, PtrbHLH, of *Poncirus trifoliata* confers cold tolerance and modulates peroxidase-mediated scavenging of hydrogen peroxide. *Plant Physiol.* **162**, 1178.

Karimi, M., Inzé, D. and Depicker, A. (2002) GATEWAY(TM) vectors for *Agrobacterium*-mediated plant transformation. *Trends Plant Sci*. **7**, 193-195.

Ma, N.N., Zuo, Y.Q., Liang, X.Q., Yin, B., Wang, G.D. and Meng, Q.W. (2013) The multiple stress-responsive transcription factor SlNAC1 improves the chilling tolerance of tomato. *Plant Physiol*. **149**, 474–486.

Schmittgen, T.D. and Livak, K.J. (2008) Analyzing real-time PCR data by the comparative C(T) method. *Nat. Protoc.* **3**, 1101.

Stevens, R., Buret, M., Garchery, C., Carretero, Y. and Causse, M. (2006) Technique for rapid, small-scale analysis of vitamin C levels in fruit and application to a tomato mutant collection. *J. Agr. Food. Chem*. **54**, 6159-6165.

Yoo, S.D., Cho, Y.H. and Sheen, J. (2007) *Arabidopsis* mesophyll protoplasts: a versatile cell system for transient gene expression analysis. *Nat. Protoc*. **2**, 1565-1572.

Waadt, R., Schmidt, L.K., Lohse, M., Hashimoto, K. and Bock, R. (2008) Multicolor bimolecular fluorescence complementation reveals simultaneous formation of alternative CBL/CIPK complexes in planta. *Plant J*. **56**, 505.

Walter, M., Chaban, C., Schütze, K., Batistic, O., Weckermann, K., Näke, C., Blazevic, D., Grefen, C., Schumacher, K. and Oecking, C. (2004) Visualization of protein interactions in living plant cells using bimolecular fluorescence complementation. *Plant J*. **40**, 428-438.

Zhang, Z., Wang, J., Zhang, R. and Huang, R. (2012) The ethylene response factor AtERF98 enhances tolerance to salt through the transcriptional activation of ascorbic acid synthesis in *Arabidopsis*. *Plant J*. **71**, 273-287.
